# Rumen microbiota-associated stress alleviation by creatine pyruvate in newly received cattle: a multi-omics study

**DOI:** 10.1186/s40168-026-02365-1

**Published:** 2026-03-05

**Authors:** Kang Mao, Yitian Zang, Chang Wang, Wenping Yang, Guwei Lu, Qinghua Qiu, Kehui Ouyang, Xianghui Zhao, Xiaozhen Song, Huan Liang, Lanjiao Xu, Mingren Qu, Yanjiao Li

**Affiliations:** 1 College of Animal Science and Technology, Jiangxi Agricultural University, Nanchang, 330045 China; 2 School of Basic Medical Sciences, Jiangxi Medical College, Nanchang University, Nanchang, 330006 China; 3 College of Marine and Bioengineering, Yangcheng Institute of Technology, Yancheng, 224051 China

**Keywords:** Newly received cattle, Creatine pyruvate, Rumen, Multi-omics, Rumen microbiota transplantation

## Abstract

**Background:**

Stress experienced by newly received cattle is a significant challenge in the beef industry, frequently resulting in weakened immune responses and impaired growth. The rumen microbiota is essential to host health, and its imbalance can exacerbate stress. This study investigates the mechanisms by which creatine pyruvate (CrPyr) mitigates stress in newly received cattle through multi-omics approaches, including metagenomics, metabolomics, in vitro and in vivo experiments, and rumen microbiota transplantation (RMT) in mice.

**Results:**

Our results revealed that CrPyr significantly reduces stress-related hormones (cortisol and adrenocorticotropic hormone) and inflammatory markers (IL-6, IL-1β, and TNF-α), and enhanced antioxidant capacity (SOD: 57.38 versus 46.93 U/mL, *P* < 0.05; GSH-Px: 305.87 versus 217.07 U/mL, *P* < 0.05; T-AOC: 9.62 versus 7.66 U/mL, *P* < 0.05). Metagenomic analysis demonstrated that CrPyr increased *Prevotella* abundance, a key rumen bacterium involved in volatile fatty acid (VFA) production, and enriches metabolic pathways associated with energy metabolism (ATP synthesis, and pyruvate metabolism) and antioxidant defense (glutathione metabolism, FC = 1.08, *P* < 0.05). In vitro and in vivo experiments, as well as RMT studies in mice, further validate these findings, demonstrating that CrPyr promote VFA synthesis and increased ATP production through the electron transport phosphorylation pathway.

**Conclusions:**

CrPyr modulates the abundance of ruminal *Prevotella* in transport-stressed cattle to enhance glutathione and VFA metabolism and to accelerate ATP and nucleotide synthesis, thereby alleviating stress in newly received cattle. This multimodal approach established CrPyr as an effective nutritional intervention that improves rumen function and increases livestock productivity.

Video Abstract

**Supplementary Information:**

The online version contains supplementary material available at 10.1186/s40168-026-02365-1.

## Background

In the global beef cattle industry, transporting and acclimating newly received cattle present major challenges, as multiple stressors during the receiving period significantly compromise animal health and productivity [1]. Prolonged fasting, water deprivation, environmental changes, and abrupt dietary transitions result in severe energy deficiency, which impairs immune function and frequently results in growth impairment, increased morbidity, and mortality, thereby causing considerable economic losses [2]. Calves are particularly susceptible due to their limited energy reserves and higher metabolic requirements, which increase their risk of energy depletion, hunger, and hypoglycemia during and after transportation [1, 3]. Consequently, expediting energy restoration and improving immune function during the receiving period are essential strategies for mitigating stress in newly received cattle within global production system.

The rumen microbiome ferments feed into volatile fatty acids (VFAs), which provide approximately 70% of ruminant energy; even micro shifts in community composition can alter VFA production and host metabolism [4, 5]. Consequently, maintaining the balance of the rumen microbial community is essential to ruminant health. Transport-induced microbial dysbiosis emerges rapidly and may persistent throughout the post-arrival period, thereby exacerbating stress-related dysfunction. Within 6 h of road transport, the abundance of *Firmicutes* (primarily *Lactobacillus*) increase while *Bacteroidetes* (primarily *Prevotella*) decrease, accompanied by elevated concentrations of lipopolysaccharide (LPS) and pro-inflammatory cytokine [6, 7]. Metagenomic profiling further indicates that the peak of systemic oxidative stress and inflammation (day 16 post-transport) coincides with a proliferation of methanogens (*Methanobrevibacter*) and pathogenic bacteria (*Saccharopolyspora* *rectivirgula*), along with a significant increase in ruminal l-ornithine-derived polyamines that sustain oxidative damage [8]. Additionally, the transport-induced alterations in the rumen microbiome and its metabolites not only closely correspond with host stress indices but also influence the host, thereby influencing the systemic stress response. Elevated LPS and polyamines levels penetrate the epithelial barrier, stimulating systemic inflammation, activating the HPA axis, and redirecting energy toward immune responses, thereby impeding growth processes [9–11]. Additionally, transportation stress can reduce rumen propionate and butyrate concentrations [7, 12]. Propionate is one of the primary energy sources for ruminants, while butyrate plays a vital role in maintaining the health and integrity of the rumen epithelium [13, 14]. Collectively, the rumen function not only as a target of transport stress but also as an active amplifier: microbial metabolites (methane, polyamines, and VFA) redirect host energy away from growth, maintain systemic inflammation, and thereby represent a major contributor to the overall transport-stress syndrome.

Dietary nutrition is a widely acknowledged factor in maintaining rumen microbial homeostasis. However, the exact requirements for energy, protein, and roughage in newly received cattle during the post-transport stress period remain inadequately investigated [15, 16]. Previous studies have consistently indicated that increasing dietary energy density or crude protein content improves average daily gain, while concurrently increasing the incidence of ruminal acidosis, liver abscesses, and associated disorders [17, 18]. Given that baseline nutrient requirements cannot be accurately quantified during this critical transition period, and marked increases in energy or protein supply carry substantial metabolic risks, the administration of host-compatible, rapidly metabolized, and multi-functional nutritional supplements represents a conservative and effective strategy to accelerate re-establishment of rumen homeostasis and to mitigate stress without imposing additional metabolic burden. Creatine pyruvate (CrPyr) is a novel multi-functional nutrient comprised of 40% pyruvate and 60% creatine, both of which are endogenous intermediate metabolites [19]. Following absorption, CrPyr dissociates into creatine and pyruvate, playing key regulatory roles in both host physiological homeostasis and ruminal microbial metabolism, particularly for rumen fermentation and microbial community balance. In the host, the absorbed creatine rapidly regenerates ATP through the phosphocreatine system to fulfill immediate energy requirements, whereas pyruvate enters the tricarboxylic acid (TCA) cycle to support ongoing ATP production and metabolic homeostasis [20]. In the rumen microbiota, CrPyr supplies both metabolites: pyruvate serves as a key intermediate in carbohydrate fermentation to generate ATP for microbial proliferation, while creatine slowly releases ammonia to provide a stable nitrogen source for microbial crude protein (MCP) synthesis [21, 22]. However, *Prevotella* spp. play a dual function within the rumen ecosystem, acting both as primary contributors to MCP synthesis and as keystone taxa involved in carbohydrate degradation [23, 24]. As a central glycolytic metabolite, supplemental pyruvate could be directly utilized by *Prevotella* to increase its succinate and propionate production. Simultaneously, the consistent release of ammonia from creatine can efficiently fulfill *Prevotella*’s nitrogen requirements for growth and protein synthesis. Therefore, CrPyr is proposed to selectively provide key substrates that promote *Prevotella*’s recovery and metabolic activity after stress-induced dysbiosis. Our previous research has indicated that CrPyr can increase ATP production during fatty acid β-oxidation and the citrate cycle, and that upregulating microbial protein synthesis in the rumen, can reduce oxidative stress, regulate energy metabolism, and improve rumen fermentation characteristics under heat stress conditions [21, 22]. Additionally, CrPyr reduces serum cortisol (COR) and LPS levels and promotes the restoration of the rumen microbiota, thereby mitigating transportation stress in beef cattle [25]. These results indicate that CrPyr can mitigate stress by promoting ATP synthesis and maintaining rumen homeostasis. However, it remains unclear which microbial taxa and metabolic pathways mediate CrPyr’s effects in correcting rumen flora imbalances, or how these changes in rumen microbiota and their metabolites impact host metabolism to relieve stress. Given the pivotal role of *Prevotella* in rumen function, this study specifically investigated the hypothesis that CrPyr alleviates stress by selectively enhancing *Prevotella* abundance and its associated metabolic activities, thereby improving host energy supply and antioxidant capacity.

Our prior research found that stress in newly received cattle is most severe on day 16 of the transition period [8]. Consequently, in this study, we administered 30 g/cattle/day of CrPyr before (to promote physiological reserves by supporting host energy metabolism and rumen microbial substrates, thereby reducing the initial stress induced by transport) and after transportation. Furthermore, we selected day 16 of the transition period as the research node. Using metagenomics and metabolomics, we investigated the mechanisms through which CrPyr alleviates stress in newly received cattle by regulating the rumen microbiota. These mechanisms were further validated through in vitro experiments, and rumen microbiota transplantation (RMT) experiments in mice. These findings establish a theoretical framework for CrPyr as a microbiota-targeted intervention to mitigate stress in newly received cattle.

## Methods

### Experiment 1: CrPyr in vivo intervention trial

#### Animals’ management

The transport management of newly received cattle followed the protocol established in our previous study [8]. In this study, 6-month-old male Chinese Simmental crossbred cattle (*n* = 17), raised under uniform feeding and environmental conditions, and sharing a compatible genetic background, were used. They were assigned to two treatments and ear tagged; Group 1 included eight calves with general management, and as a control group, Group 2 consisted of nine calves with drinking water containing CrPyr and as a CrPyr group. The day before transportation, the calves in the CrPyr group were administered CrPyr at a dose of 30 g/day/per calf, which was dissolved in drinking water. This dosage was based on a previous study by Liu et al. [22]. The 17 calves were transported along with 83 other 6-month-old male Chinese Simmental crossbred cattle from the Livestock and Forage Trading Market in Altay City, Xinjiang Uygur Autonomous Region, to Xuchang City, Henan Province. The journey commenced at 20:00 on 13 September 2021 and concluded at 15:00 on 16 September 2021, lasting 67 h and covering approximately 3450 km on highways and urban roads at a maximum speed of 70 km/h. Prior to transport, calves were fed a diet consisting of 30% concentrate and 70% wheat straw (DM basis). The concentrate was obtained from a commercial company, and contained crude protein ≥ 17%, crude fiber ≤ 10%, crude ash ≤ 9%, moisture ≤ 14%, total phosphorus ≥ 0.4%, calcium 0.5 − 1.2%, salt 0.8 − 1.5%. Following commingling, all calves were transported via a double-deck livestock trailer (Zhumadian CIMC Huajun Vehicle Co., Ltd, China; 13.5 m length × 2.3 m width × 4.2 m height) equipped with quilts and tarpaulins for insulation. Experimental calves were confined to the upper deck. During transit, cattle were deprived of water; however, they had access to wheat straw, which was placed on the sides of the cart in the form of straw bundles during loading. Upon arrival, the 17 calves were confined in the same barn and assigned to their respective treatment groups according to pre-assigned ear-tag numbers. The calves in the treatment group continued to receive 30 g/day/calf of CrPyr through drinking water for 16 days post-arrival, while the control group remained untreated [8]. During the initial 2 days of the receiving period, calves were predominantly fed with wheat straw. Subsequently, calves were transitioned to a total mixed ration (TMR). The composition and nutrient levels of the TMR are provided in Table S1, which was provided twice daily. Water intake for beef cattle was strictly controlled for the first five days after arrival. The water temperature was about 37 °C, and it was administered three times a day, with approximately 3 L each time. In subsequent experiments, water at normal temperature was provided freely.

#### Sample collection

On day 16 post-arrival, 10 mL of whole blood was aseptically collected from the jugular vein into serum-separator tubes without anticoagulant. After centrifugation at 3000 × g for 15 min at 4 °C, the harvested serum was snap-frozen in liquid nitrogen and stored at − 80 °C until analysis. Additionally, rumen fluid was obtained through an oral stomach tube; the tubing was washed thoroughly with running water between animals, and the first 50 mL were discarded to reduce salivary contamination. Immediately thereafter, pH of 50 mL of fresh rumen fluid was measured using a portable pH meter (HANNA Instruments, Cluj-Napoca, Romania). The remaining fluid was aliquoted into sterile cryovials, snap-frozen in liquid nitrogen, and stored at − 80 °C for subsequent analyses.

#### Chemical analyses

The concentrations of serum COR, (catalog numbers: H094-1-2, intra-assay CV ≤ 8%, inter-assay CV ≤ 10%), adrenocorticotropic hormone (ACTH, catalog numbers: H097-1-2, intra-assay CV ≤ 8%, inter-assay CV ≤ 10%), total antioxidant capacity (T-AOC, catalog numbers: A015-2-1), superoxide dismutase (SOD, catalog numbers: A001-3-1), glutathione peroxidase (GSH-PX, catalog numbers: A005-1-2), malondialdehyde (MDA, catalog numbers: A003-1-2), IgA, IgG, IgM (catalog numbers: H108-1-2, H106-1-1, H109-1-2, intra-assay CV ≤ 8%, inter-assay CV ≤ 10%), interleukin-1β (IL-1β, catalog numbers: H002-1-2, intra-assay CV ≤ 8%, inter-assay CV ≤ 10%), IL-4 (catalog numbers: H005-1-2, intra-assay CV ≤ 8%, inter-assay CV ≤ 10%), IL-6 (catalog numbers: H007-1-2, intra-assay CV ≤ 8%, inter-assay CV ≤ 10%), tumor necrosis factor-α (TNF-α, catalog numbers: H052-1-2, intra-assay CV ≤ 8%, inter-assay CV ≤ 10%) and rumen ATP (catalog numbers: A095-1-1), were measured using commercial assay kits from Nanjing Jiancheng Bioengineering Institute (Nanjing, China) following the manufacturer’s instructions. The VFA concentrations in the rumen fluid samples were determined by gas chromatography (Shimadzu GC-2014, Japan) equipped with a capillary column (Stabilwax, Restek, Bellefonte, PA, USA), following the procedures and parameter settings outlined by Qiu et al. [26]. Briefly, ruminal fluid (800 µL) was deproteinized with 200 µL internal standard (25% metaphosphoric acid + 2-ethylbutyric acid 2.17 mL L^−1^). After 30 min on ice and centrifugation (12,000 × g, 10 min, 4 °C), 200 µL of the supernatant was injected into a GC-2014 equipped with an Rtx-Wax column (30 m × 0.25 mm × 0.25 µm) and FID. The oven programme was set to 110 °C for 30 s, increased to 120 °C at 10 °C min^−1^, held for 4 min. Then, the temperature was raised to 150 °C at 10 °C min^−1^ and held for 3 min. Carrier N_2_ was supplied at a flow rate of 2.5 mL min^−1^ with a split 20:1.

#### Rumen metagenomics

Metagenomic DNA extraction, library construction, and sequencing were performed as described previously [8]. Briefly, total genomic DNA was extracted from rumen fluid samples (*n* = 6 for each group) using the FastDNATM Spin Kit for Stool (MP Biomedicals, USA). The concentration and purity of the extracted DNA were subsequently determined through the TBS-380 fluorometer and the NanoDrop2000 spectrophotometer, respectively. For paired-end library construction, DNA was fragmented to an average size of about 400 bp using Covaris M220 (Gene Company Limited, China). Paired-end sequencing was performed on Illumina NovaSeq 6000 (Illumina Inc., San Diego, CA, USA) at Majorbio Bio-Pharm Technology Co., Ltd. (Shanghai, China). Sequence data associated with this project have been deposited in the NCBI Short Read Archive database (BioProject ID: PRJNA1310242).

The raw metagenomic sequencing data were processed to obtain high-quality clean reads by filtering out low-quality reads (quality scores < 20 or length < 50 bp or having N bases) using the fastp tool [27] (https://github.com/OpenGene/fastp, version 0.20.0). The reads were aligned to the bovine genome (bosTau8 3.7, 10.18129/B9.bioc.BSgenome.Btaurus.UCSC.bosTau8) using BWA (http://bio-bwa.sourceforge.net) to filter out host DNA [28]. The filtered reads were de novo assembled for each sample using Megahit [29] (https://github.com/voutcn/megahit, version 1.1.2). Contigs with a length ≥ 300 bp in length were selected as the final assembly result. Open reading frames (ORFs) from each assembled contig were predicted using MetaGene [30] (http://metagene.cb.k.u-tokyo.ac.jp/), and a length of ≥ 100 bp was retrieved. Non-redundant contigs were identified using CD-HIT [31] (http://www.bioinformatics.org/cd-hit/, version 4.6.1) with 90% sequence identity and 90% coverage. The quality-filtered sequence reads were mapped to the representative sequences with 95% identity using SOAPaligne [32] (http://soap.genomics.org.cn/, version 2.21). The gene abundance in each sample was calculated as reads per kilobase per million mapped reads (RPKM).

The best-hit taxonomy of non-redundant genes was determined by aligning them against the NCBI NR database using Diamond [33] (http://ab.inf.uni-tuebingen.de/software/diamond/, version 2.0.13) with an *e* value cut-off of 1e^−5^. Feature abundance was normalized using the relative abundance of each sample. Microbial taxa with average relative abundances > 0.01% were used for downstream analysis. The Kyoto encyclopedia of genes and genomes (KEGG) annotation was performed using Diamond [33] against the KEGG database (http://www.genome.jp/keeg/, version 94.2). Additionally, RPKM with average relative abundances > 100 were used for downstream analysis. All these databases used an *E* value cut-of 1e^−5^ for ORF annotation. The differential analysis was performed at each taxonomic, functional, or gene-wise level using the Kruskal–Wallis test.

#### Rumen metabolomics

The rumen samples were analyzed using the LC–MS platform (Thermo, UHPLC -Q Exactive HF-X).

#### Metabolite extraction

Metabolites were extracted from rumen fluid and serum using the method described previously [8]. Briefly, 100 µL of the liquid sample was aspirated, and the metabolites were extracted with 400 µL of a methanol:water (4:1, v/v) solution. The mixture was incubated at − 20 °C and treated with a high-throughput tissue crusher (Wonbio-96c, Shanghai Wanbo Biotechnology Co., LTD) at 50 Hz for 6 min, followed by vortexing for 30 s and ultrasound at 40 kHz for 30 min at 5 °C. The samples were placed at − 20 °C for 30 min to precipitate proteins. After centrifugation at 13,000 × g at 4 °C for 15 min, the supernatant was carefully transferred to sample vials for LC–MS/MS analysis.

#### Metabolomics data analysis

LC–MS data were analyzed using Progenesis QI 2.3 (Waters Corporation, Milford, USA) to extract raw peaks, filter and calibrate the baseline, align, deconvolute, identify peaks, and integrate peak areas. Rumen and serum metabolites that were present in < 50% of samples or with a relative standard deviation > 30% were removed. Following normalization procedures and imputation, statistical analysis was performed on log-transformed data to identify significant differences in metabolite levels between comparable groups. The analysis of metabolite sources was performed in MetOrigin [34] (https://metorigin.met-bioinformatics.cn). A multivariate statistical analysis was performed using ropls (http://bioconductor.org/packages/release/bioc/html/ropls.html, version 1.6.2) R package from Bioconductor on Majorbio Cloud Platform (https://cloud.majorbio.com). Partial least squares discriminate analysis (PLS-DA) and *t* test were performed between control and CrPyr groups, with *P* values < 0.05 and the VIP > 1 indicating significantly different metabolites. The enrichment analysis performed in MetOrigin was applied to each metabolite from each cluster to identify metabolic pathways (*P* < 0.05) [34]. The metabolomics data have been deposited in the National Center for Bioinformation under accession number OMIX013787.

### Experiment 2: CrPyr in vitro validation trial

#### The construction of the in vitro rumen fermentation model and experimental designs

All rumen fluid donor animals were kept in Minghui Agricultural Co., Ltd., a beef cattle breeding facility located in Yongxin County, Ji’an City, Jiangxi Province, China. The experimental design is illustrated in Fig. 5A.

#### The donor animal’s management

The administrator of Minghui Agricultural Company obtained 100 6-month-old male Chinese Simmental crossbred calves, with similar genetic backgrounds and raised under identical feeding and environmental conditions before sale, from the Livestock and Forage Trading Market in Altay City, Xinjiang Uygur Autonomous Region. Following unique ear individualized tagging, all calves were randomly divided into two adjoining pens (Pen 1 and 2) and kept in the same barn for 3 days. The diet (70% straw and 30% concentrate) was provided twice daily at 8:00 AM and 4:00 PM, with free access to water. After the feeding period, the calves were transferred to a double-decker truck (13.5 m long, 2.3 m wide, and 4.2 m high) filled with hay in preparation for transportation to the company’s feedlot. Calves from Pen 1 were placed on the upper deck, while those from Pen 2 were placed on the lower deck. The travelling distance was 4025 km. During the approximately 70 h of transportation, the calves were deprived of water; however, they had access to a limited amount of hay. Upon arrival, the calves were assigned to four pens based on their position during transportation: calves from the upper deck were placed in Pens A and B, while those from the lower deck were placed in Pens C and D, with 25 calves in each pen. During the initial 3 days, the calves were primarily fed straw to acclimate to the new environment. After the acclimation period, the calves were provided with a TMR composed of straw, Napier grass, and concentrate, with the specific composition and nutritional levels of the TMR detailed in Supplementary Table 2. Following arrival, CrPyr was added to the drinking water at a dosage of 30 g/day/cattle in Pen A and C. This dosage was based on the study by Liu et al. [22].

#### Rumen fluid collection

On day 15 after arrival, six calves (three from each group) were randomly selected from Pens A, B, C and D for rumen fluid collection through oral sampling. Pens A and C served as the treatment group, while Pen B and D were in the control group. The collected rumen fluid was filtered through four layers of gauze, pooled, and stored in sterile thermos flasks to serve as the inoculum for the in vitro fermentation trial. After collection, the samples were immediately transported to the laboratory, where the pH was measured using a PHBJ-260 pH meter (Shanghai INESA Scientific Instrument Co., Ltd., Shanghai, China), with averaged values of 6.82 and 6.79, respectively. The fermentation substrate was consistent with the farm’s diet (Supplementary Table 2).

#### Experimental design and in vitro cultivation medium

The experiment was divided into two groups: a control group and a CrPyr treatment group, each with 14 replicates. Following 24 h of fermentation, the samples were placed on ice for 30 min to terminate fermentation. All parameters were measured strictly in accordance with the experimental procedures and requirements. The preparation of the mixed culture medium was based on the study by Mao et al. [35], with the specific formulation ratios detailed in Supplementary Table 3. For the treatment group, an additional 300 µL of 0.1 g/mL CrPyr solution was added, with the dosage determined based on the amount used in the rumen fluid donor animals. The bottles were incubated anaerobically in a 39 °C SHA-B shaker (Guohua Enterprise, Changzhou, Jiangsu, China) for the in vitro rumen fermentation experiment.

#### Chemical analyses and metagenomic procedures

Rumen fermentation parameters (VFA, NH_3_-N, and MCP) and metagenomic procedures (DNA extraction, library preparation, and shotgun sequencing) were performed as described for Experiment 1. Raw sequence data have been deposited in the NCBI Sequence Read Archive under BioProject ID PRJNA1310418.

### Rumen energy metabolomics

#### Metabolite extraction

Rumen fluid (100 µL) was thoroughly mixed with 400 µL of cold methanol acetonitrile (v/v, 1:1) through vortexing. Then the mixtures were sonicated for 1 h in an ice bath, followed by incubation at − 20 °C for 1 h, and centrifuged at 4 °C for 20 min with a speed of 14,000 × g. The supernatants were then harvested and dried under vacuum LC–MS analysis.

Furthermore, to maintain data quality for metabolic profiling, quality control (QC) samples were prepared by pooling aliquots of all samples, representing the entire dataset, and used for data normalization. QC samples were prepared and analyzed using the same procedure as for the experimental samples in each batch. Dried extracts were then dissolved in 50% acetonitrile. Each sample was filtered with a disposable 0.22 µm cellulose acetate and transferred into 2 mL HPLC vials and stored at − 80 °C until analysis.

#### UHPLC-MS analysis

The LC/MS portion of the platform was based on a Shimadzu Nexera X2 LC-30AD system equipped with an ACQUITY UPLC BEH Amide column (1.7 μm, 2.1 mm × 100 mm, Wasters) and a triple quadruple mass spectrometer (QTRAP 5500, AB SCIEX). Metabolites were detected in electrospray negative-ionization and positive-ionization modes. For each sample, 2 μL was injected sequentially into the LC autosampler. The ACQUITY UPLC BEH Amide column (1.7 μm, 2.1 × 100 mm, Wasters) was heated to 45 °C at a flow rate of 300 μL/min. A gradient was used to separate the compounds consisting of 20 mM ammonium acetate and 5% acetonitrile with pH 9.45 (solvent A) and 100% acetonitrile (solvent B). The gradient started at 5% solvent A for 1 min and increased linearly to 45% solvent A over 12 min. Subsequently, it increased linearly to 60% solvent A over 1 min with a 2-min hold before returning to the initial mixture during 0.1 min and re-equilibrating for 3 min. QC samples were injected after every eight samples during acquisition.

MS conditions were set as follows: Source Temperature 550 ℃, Ion Source Gas1 (GAS1): 40, Ion Source Gas2 (GAS2): 50, Curtain Gas (CUR): 35, and Ion Spray Voltage Floating (ISVF): − 4500 V. The mass spectrometer was operated with a 200 ms dwell time. To construct the metabolite multiple reaction monitoring (MRM) library, each metabolite standard (50 mg/mL) was first analyzed by LC–MS/MS to obtain the optimal MRM transition parameters. Then, the retention times of 40 energy-related metabolites were determined by measuring their corresponding MRM (Q1/Q3) transitions individually. A serial dilution of the reference standard, including 40 energy-related metabolites, was prepared for LC–MS analysis. The calibration curve was constructed from the peak areas of each standard metabolite in the serially diluted reference standard solution, using the least-squares method to determine the corresponding concentration. The calibration curves, linearities, and correlation coefficients (R^2^) are provided in Supplementary Table 4. The calibration curve with an R^2^ > 0.99 was acceptable. The quantitative concentration of each metabolite was calculated from the calibration curve.

### Experiment 3, rumen microbiota transplantation validation trial

#### RMT in mice

Rumen fluid inoculation was conducted according to protocols previously described, with minor modifications [36, 37]. The rumen fluid collected in Experiment 1 was used as the transplant inoculum. Briefly, a sterilized, weighted oral stomach tube (1.2 m length, 10 mm i.d., PVC) was passed into the rumen in newly received cattle; the first 50 mL was discarded to remove saliva. A 200 mL aliquot was collected per calf into pre-warmed (39 °C), CO_2_-flushed, insulated thermos flasks. Samples were pooled (equal volume from each calf) to create one composite inoculum. Large feed particles were removed by squeezing through two layers of sterile cheesecloth followed by centrifugation at 400 × g, 4 °C, for 5 min. The supernatant was re-centrifuged at 6000 × g, 4 °C, for 5 min to harvest microbial. The supernatant was discarded, and the pellet was resuspended in sterile 1 × PBS. The resulting suspension was immediately administered to recipient mice. Thirty male Kunming mice (age 6 weeks) were adapted for 7 days and then subjected to a broad-spectrum antibiotic cocktail (ampicillin 1 g/L, ciprofloxacin 0.2 g/L, metronidazole 1 g/L) by oral gavage for seven consecutive days to deplete the gut microbiota [38]. Mice were subsequently randomized into three groups (*n* = 10 per group) and treated daily for 14 days: (i) CON, 200 µL saline; (ii) rumen microbiota transplantation (RMT), 200 µL rumen fluid; (iii) RMT_CrPyr, 200 µL rumen fluid and 300 µL CrPyr (0.1 g/mL). On day 14, mice were euthanized under isoflurane anesthesia. Blood was collected by retro-orbital puncture into serum-separating tubes without anticoagulant, then centrifuged at 3000 × g for 15 min at 4 °C. The serum was stored at − 80 °C for targeted energy metabolomics. Colonic contents were snap-frozen in liquid nitrogen and stored at − 80 °C for metagenomic sequencing, using the same procedures as in Experiments 1 and 2. Sequence data associated with this project have been deposited in the NCBI Short Read Archive database (BioProject ID: PRJNA1320625).

#### Statistical analysis

For Experiments 1 and 2, chemical variables were compared between the two groups using *t* tests in SPSS (version 17.0, IBM, Armonk, NY, USA). In Experiment 3, differences among multiple groups were determined by using one-way ANOVA followed by Duncan’s post-hoc test. A two-tailed *P* value < 0.05 was considered statistically significant. For metagenomic analyses, differential features in Experiments 1 and 2 were identified by the Wilcoxon rank-sum test, with significance defined as an FDR-adjusted *P* < 0.05. In Experiment 3, differentially abundant taxa were determined using linear discriminant analysis effect size (LEfSe), with a linear discriminant analysis (LDA) score threshold > 2 and a *P* value < 0.05.

## Results

### Experiment 1:The experimental design is illustrated in The experimental design is illustrated in effects of CrPyr on the host physiology and ruminal microbiota of newly received cattle

#### Serum parameters of newly received cattle

**Fig. 1:**
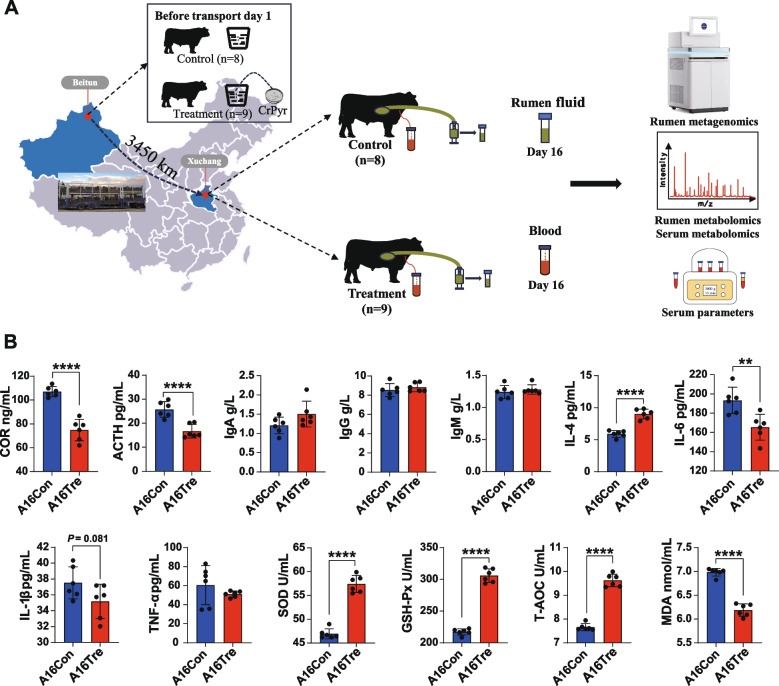
Schematic diagram of the experimental design and serum indices of newly received cattle. **A** Schematic diagram of the experimental design and procedures in this study. **B** Serum hormone indices, immunoglobulin factors, inflammatory factors, and antioxidant parameters of newly received cattle. A16Con control group at day 16 after transportation, A16Tre CrPyr treatment group at day 16 after transportation. A Student’s *t* test was used to determine the differences between the two groups. **P < 0.01, ***P < 0.001, ****P < 0.0001. *n* = 6

The experimental design is illustrated in Fig. 1A. To evaluate the effect of CrPyr in newly received cattle, we quantified serum stress-related hormones, immunoglobulins, antioxidant parameters, and inflammatory mediators (Fig. 1B). CrPyr significantly reduced COR and ACTH concentrations (*P* < 0.05); however, it exhibited no significant effect on IgA, IgG, or IgM levels (*P* > 0.05). Regarding inflammatory responses, CrPyr significantly upregulated the anti-inflammatory cytokine IL-4 while simultaneously suppressing pro-inflammatory cytokines IL-6, IL-1β, and TNF-α (*P* < 0.05). Additionally, CrPyr enhanced the activities of SOD and GSH-Px and increased T-AOC, whereas MDA content was significantly decreased (*P* < 0.05).

#### Profiling of the rumen metagenomics

**Fig. 2:**
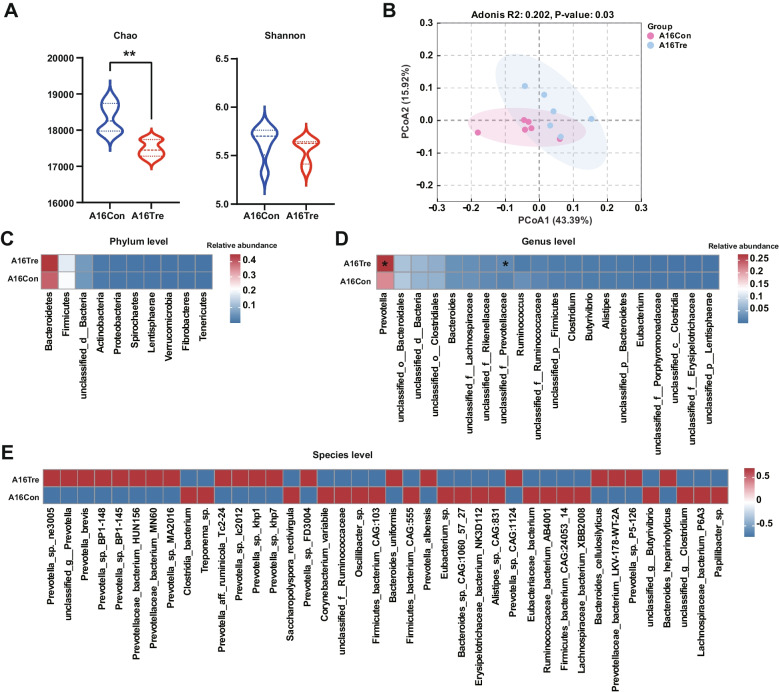
Microbial compositional profiles of newly received cattle on day 16. **A** Alpha diversity at the bacterial species level. **B** Principal coordinate analysis (PCoA) of the rumen bacterial community between the two groups. The *P* value was tested using Adonis. **C** Relative abundances of the top 10 bacterial phyla. **D** Relative abundances of the top 20 bacterial genera. **E** The top 40 bacterial species with significant differences in relative abundance at the species level. A16Con control group at day 16 after transportation, A16Tre CrPyr treatment group at day 16 after transportation. Differences in bacterial abundance were analyzed using the Wilcoxon rank-sum test. *P < 0.05. *n* = 6

Metagenome sequencing generated a total of 963,200,860 reads, with 80,266,738.33 ± 1,931,753.40 reads (mean ± standard error of the mean [SEM]) per sample (Table S5). After QC and removing host genes, a total of 936,889,050 reads were retained, with 78,074,087.5 ± 1,898,740.73 per sample. Following de novo assembly, a total of 9,937,911 contigs were generated (the N50 length of 780 ± 14.60 bp), with 828,159.25 ± 30,585.97 per sample. The rumen metagenome consisted of 98.18% bacteria, 1.03% archaea, 0.44% eukaryotes, 0.19% viruses, and 0.15% unclassified bacteria (Fig. S1). Subsequent analyses focused exclusively on the bacterial fraction. At the species level, the A16Tre group exhibited a significantly lower Chao richness compared with the A16Con group (Fig. 2A; *P* < 0.05). Principal coordinate analysis (PCoA) based on the Bray–Curtis dissimilarity index demonstrated that distinct discrimination of microbial composition between the two groups (Fig. 2B; PERMANOVA, *P* = 0.03).

At the phylum level, the ruminal bacterial communities did not differ significantly between the two groups (Fig. 2C; *P* > 0.05). Among the 20 most abundant genera, *Prevotella* was enriched in the CrPyr group compared with the control (Fig. 2D; *P* < 0.05). *Prevotella* is a predominant ruminal genus that plays a pivotal role in VFA production [39], suggesting that CrPyr treatment accelerates the re-establishment of key bacterial taxa by day 16 post-arrival in newly received cattle. At the species level, a total of 126 differentially abundant species were identified (Table S6). Among the 40 most prevalent species, 20 exhibited a significantly higher abundance in the A16Tre group (Fig. 2E), which including 13 *Prevotella *sp., 3 *Prevotellaceae* sp., 3 *Bacteroides *sp., and 1 unclassified taxon. The remaining 20 species were enriched in the A16Con group.

#### Functional analysis of the metagenomics

**Fig. 3:**
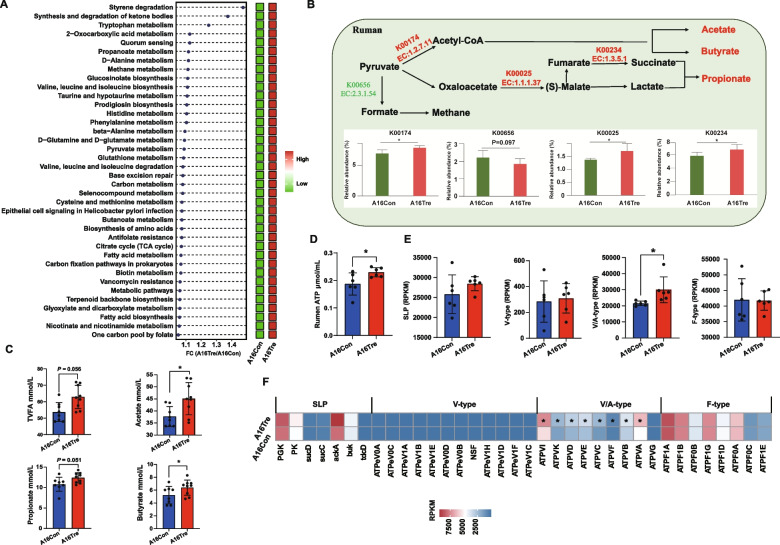
Differential KEGG functions of rumen bacteria between the two groups. **A** Significantly different KEGG pathways. **B** Metabolic pathways involved in the biosynthesis of volatile fatty acids (VFAs). **C** Concentrations of major volatile fatty acids (VFAs) in the rumen between the two groups. **D** Concentrations of rumen ATP. **E** Total abundance of substrate-level phosphorylation (SLP) enzymes and ATPases. **F** Heatmap of the abundance of SLP enzymes and F-type, V/A-type, and V-type ATPases. A16Con control group at day 16 after transportation, A16Tre CrPyr treatment group at day 16 after transportation. Differences in KEGG pathways were analyzed using the Wilcoxon rank-sum test. *P < 0.05. *n* = 6–9

To explore the functional differences in the rumen microbiota between the two groups, KEGG enrichment analysis was performed using metagenomic data. A total of 215 pathways were identified (Table S7). The pathways exhibiting significant differential abundance are depicted in Fig. 3A. Among these, 38 pathways were significantly enriched in the A16Tre group. The majority were involved in carbohydrate metabolism pathways and amino acid metabolism pathways. Specifically, carbohydrate-related pathways included pyruvate metabolism (*P* = 0.026), TCA cycle (*P* = 0.015), butanoate metabolism (*P* = 0.008), and propanoate metabolism (*P* = 0.004). Amino-acid-associated pathways comprised d-glutamine and d-glutamate metabolism (*P* = 0.008), d-alanine metabolism (*P* = 0.002), β-alanine metabolism (*P* = 0.041), cysteine and methionine metabolism (*P* = 0.004), histidine metabolism (*P* = 0.025), valine, leucine, and isoleucine biosynthesis (*P* = 0.015), phenylalanine metabolism (*P* = 0.041), valine, leucine, and isoleucine degradation (*P* = 0.043), and glutathione metabolism (*P* = 0.002). Subsequently, key genes within the carbohydrate-metabolizing pathways governing VFA biosynthesis were examined. The relative abundances of K00174 (*korA*, *oorA*, and *oforA*; catalysing the conversion of pyruvate to acetyl-CoA), K00025 (*MDH1*; mediating the reduction of oxaloacetate to malate), and K00234 (*SDHA*, and *SDH1*; driving the reduction of fumarate to succinate) were all significantly elevated in the A16Tre group (Fig. 3B; *P* < 0.05). Concordantly, ruminal VFA concentrations exhibited a parallel increase, with acetate, propionate, and butyrate all being higher in the A16Tre group compared to the A16Con group (Fig. 3C; *P* < 0.05).

Considering that CrPyr is a pivotal intermediate in energy metabolism, we next investigated its influence on ruminal ATP synthesis. Quantification of rumen ATP revealed that CrPyr supplementation elevated ATP concentrations in newly received cattle on day 16 (Fig. 3D; *P* < 0.05), indicating that CrPyr directly promotes microbial ATP synthesis in the rumen. ATP is primarily generated through substrate-level phosphorylation (SLP) and electron-transport phosphorylation (ETP). To investigate the underlying molecular mechanisms, we profiled microbial genes associated with ATP formation. In total, 7 genes encoding SLP-related enzymes and 34 genes encoding ETP ATP synthases, comprising F-, V/A-, and V-type sub-classes, were identified (Table S8). Differential analysis indicated that the relative abundance of V/A-type ATP synthase were significantly higher in the A16Tre group (Fig. 3E; *P* < 0.05), primarily attributed to increased expression of *ATPVI*, *ATPVK*, *ATPV1D*, *ATPVE*, *ATPVC*, *ATPVF*, *ATPVB*, and *ATPA* (Fig. 3F). Collectively, these results suggest that CrPyr enhances ruminal ATP synthesis in newly received cattle, at least in part, by upregulating V/A-type ATP synthase-mediated ETP.

#### LC/MS analysis of rumen metabolomics

**Fig. 4:**
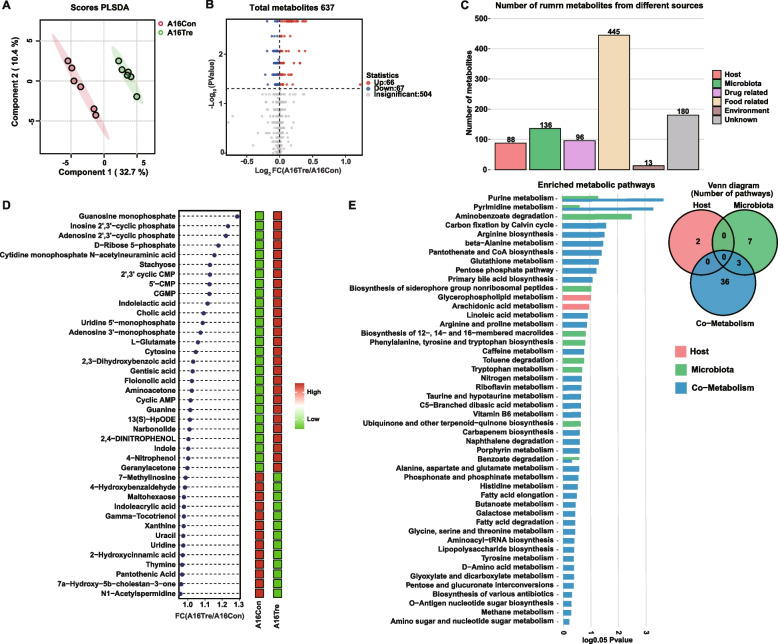
Rumen metabolome of newly received cattle between the two groups. **A** Partial least squares-discriminant analysis (PLS-DA) of the rumen metabolome. **B** Volcano plot of metabolites identified by the rumen metabolome. **C** Number of metabolites from different sources. **D** Differential metabolites originating from the host, microbiota, or both. **E** Metabolic pathway enrichment analysis according to different categories of metabolites belonging to the host, bacteria, or both. A16Con control group at day 16 after transportation, A16Tre CrPyr treatment group at day 16 after transportation. Differences in KEGG pathways were analyzed using the Wilcoxon rank-sum test. *P < 0.05. *n* = 6

After rigorous quality screening and identification, 637 reliable metabolites were identified in the rumen fluid samples of the two groups (Table S9). The results of the partial least squares-discriminant analysis (PLSDA) showed distinct clusters of metabolomes (Fig. 4A). Additionally, 133 differential metabolites were identified between the two groups (Fig. 4 and Table S9;* P* < 0.05). Metabolites source annotation identified 88 host-derived metabolites, 136 microbiota-derived, 96 drug-related, 445 food-related, 13 environmental-derived, and 180 from unknown sources (Fig. 4C and Table S9). Comprehensive profiling of host- and microbiota-derived metabolites identified 45 significantly altered compounds (Fig. 4D and Table S10; *P* < 0.05). These differential metabolites were enriched in 8 KEGG pathways, including purine metabolism, pyrimidine metabolism, arginine biosynthesis, β-alanine metabolism, pantothenate and CoA biosynthesis, glutathione metabolism, the pentose-phosphate pathway, and primary bile-acid biosynthesis (Fig. 4E; *P* < 0.05). Comparative integration with metagenomic KEGG pathway data revealed two concomitantly perturbed pathways—β-alanine metabolism and glutathione metabolism—each pathway 2 metabolites: uracil (FC = 0.978, *P* = 0.008), pantothenic acid (FC = 0.972, *P* = 0.041), and l-glutamate (FC = 1.259, *P* = 0.026), 2′,3′-cyclic CMP (FC = 1.133, *P* = 0.002). Microbial gene expression analysis indicated that genes encoding enzymes involved in l-glutamate metabolism (K00261: glutamate dehydrogenase [EC:1.4.1.3]; K06048: glutamate-cysteine ligase [EC:6.3.2.2]) were significantly upregulated in the treated group (Fig. S2, *P* < 0.05). These gene expression changes were closely correlated with the elevated l-glutamate levels, suggesting that microbes improved glutathione synthesis capacity by upregulating these genes.

### Experiment 2: effects of CrPyr on the composition of rumen microbiota in newly received cattle in vitro fermentation conditions

**Fig. 5:**
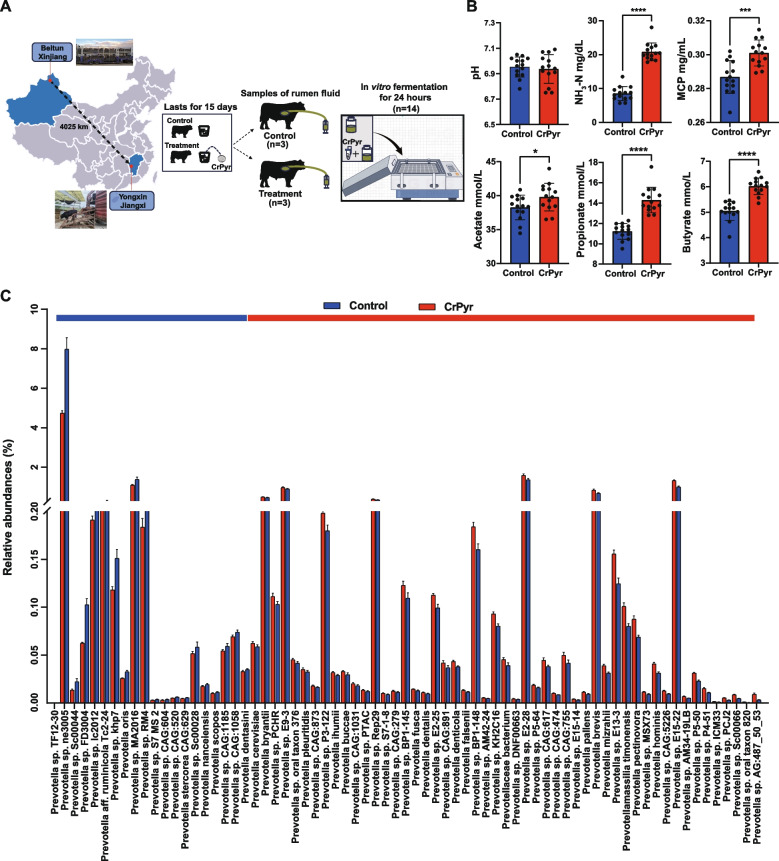
Effects of CrPyr on fermentation parameters and *Prevotella* species in an in vitro rumen fermentation model of newly received cattle. **A** Experimental design of the in vitro fermentation model. **B** Changes in rumen fermentation parameters, including pH, NH_3_-N, microbial crude protein (MCP), and volatile fatty acids (VFAs). **C** *Prevotella* species with significant differences at the species level. Differences in rumen fermentation parameters were analyzed using a *t* test, while differences in *Prevotella* species were analyzed using the Wilcoxon rank-sum test. *P < 0.05, **P < 0.01, ***P < 0.001, ****P < 0.0001. *n* = 8–14

The experiment was conducted in a completely randomized design (Fig. 5A). Rumen fermentation parameters were first quantified, revealing that CrPyr significantly elevated NH_3_-N, MCP, acetate, propionate, and butyrate concentrations compared to the control (Fig. 5B; *P* < 0.05). These data collectively indicate that CrPyr improves the in vitro ruminal fermentation profile of newly received cattle.

#### Profiling of the rumen metagenomics in vitro fermentation

Metagenome sequencing generated a total of 686,743,154 reads, with 42,921,447 ± 989,108 reads (mean ± standard error of the mean [SEM]) per sample (Table S11). After QC and removal of host genes, a total of 679,331,776 reads were retained, averaging 42,458,236 ± 971,189 reads per sample. After de novo assembly, a total of 5,542,486 contigs were generated (the N50 length of 1069 ± 27), with an average of 346,405 ± 11,094 contigs per sample.

The microbial domains of the rumen microbiomes were compared between of the two groups. The composition of bacteria, archaea, and viruses exhibited significant differences (Fig. S3A, *P* < 0.05). However, given that archaea and viral sequences constitute only a small fraction of the rumen metagenome, statistical analysis primarily focused on bacterial composition. Consistent with these findings, significant differences were detected in the Chao and Shannon indices of rumen bacteria between the two groups (Fig. S3B;* P* < 0.05). Furthermore, the PCoA based on Bray–Curtis revealed distinct separations in the species-level composition of rumen bacteria (Fig. S3D; PERMANOVA, *P* = 0.001) between the two groups.

At the phylum level, differential abundance analysis of the top 9 most abundant phyla revealed significant differences in five phyla. Specifically, *Proteobacteria* exhibited a significant decrease, while *Firmicutes*, *Actinobacteria*, *Tenericutes*, and *Candidatus Saccharibacteria* were significantly enriched in the CrPyr group (Fig. S3D, *P* < 0.05). Among the top 20 most abundant genera, 14 bacterial genera showed significant differences. 10 genera, including *Bacteroides*, *Clostridium*, *Butyrivibrio*, *Alistipes*, *Eubacterium*, *Candidatus Nanosyncoccus*, *Oribacterium*, *Parabacteroides*, *Lachnoclostridium*, and *Phocaeicola*, were significantly enriched in the CrPyr group. In contrast, 4 genera, including *Ruminobacter*, *Succinivibrio*, *Pseudobutyrivibrio*, and *Selenomonas*, were significantly reduced in the CrPyr group (Fig. S3E, *P* < 0.05). At the species level, a total of 175 *Prevotella *sp. were identified (Table S12). Among them, 100 species exhibited significant differences in abundance between groups, as assessed by Wilcoxon rank-sum test (Table S13;* P* < 0.05). Within the 71 most abundant differential *Prevotella *sp., 51 were enriched in the CrPyr group, whereas 20 were enriched in the control group (Fig. 5C; *P* < 0.05). Subsequent comparison of these differential *Prevotella *sp. with the taxa that were altered in vivo revealed 13 concordantly responsive species, 6 of which included—*Prevotella brevis*, *Prevotella *sp.* P5*-*50*, *Prevotella *sp.* P3*-*120*, *Prevotella *sp.* BP1*-*145*, *Prevotella *sp.* BP1*-*148*, and *Prevotella *sp.* AM42-24*—were significantly enriched in the CrPyr-treated group (Fig. S3).

#### Rumen microbial functions as determined by metagenomics in vitro fermentation

**Fig. 6:**
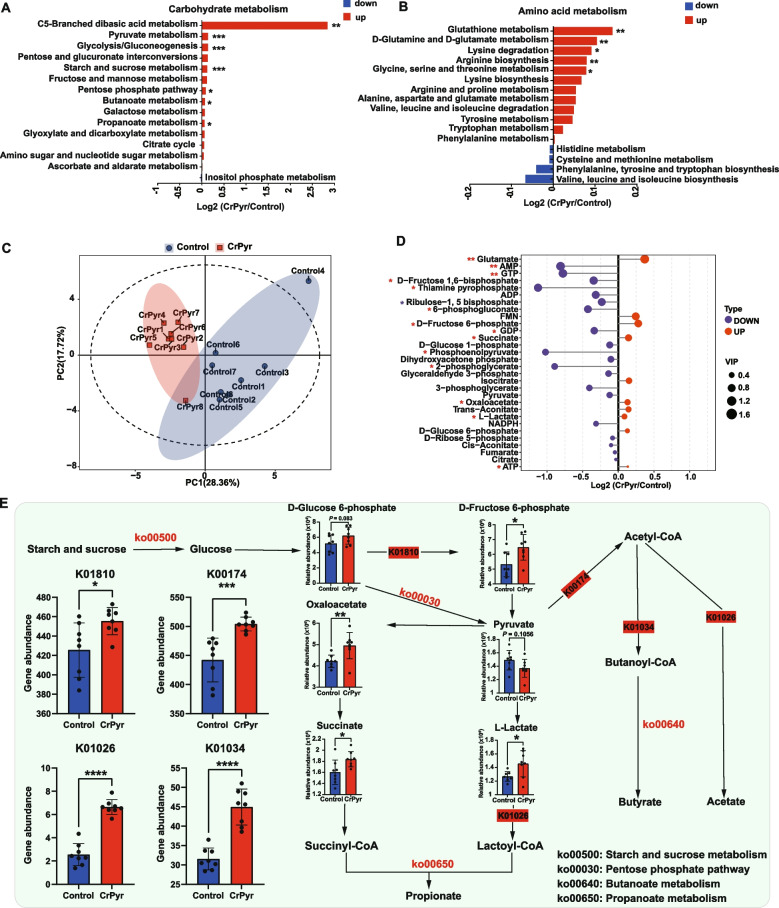
KEGG functional analysis and differential metabolites in the in vitro rumen fermentation model of newly received cattle. **A** Carbohydrate metabolism at KEGG level 3. **B** Amino acid metabolism at KEGG level 3. **C** Principal component analysis (PCA) of targeted energy metabolomics. **D** Differential metabolites between the control and CrPyr groups. **E** Metabolic pathways, metabolites, and KOs involved in the synthesis of volatile fatty acids (VFAs). Differences were analyzed using the Wilcoxon rank-sum test. *P < 0.05, **P < 0.01, ***P < 0.001, ****P < 0.0001. *n* = 8

Functional annotation based on KEGG profiling 278 endogenous third-level pathways, which were considered as rumen microbial metabolic pathways (Table S14). These pathways belonged to six first-level categories, including Metabolism (63.61 ± 2.32%), Genetic Information Processing (18.33 ± 0.61%), Cellular Processes (6.52 ± 0.65%), Human Diseases (5.39% ± 0.63%), Organismal Systems (3.51 ± 1.01%), and Environmental Information Processing (2.63 ± 0.54%). Notably, “Metabolism” exhibited significant enrichment in the rumen microbiome of the CrPyr group compared to the control group (Fig. S3F;* P* < 0.05). At KEGG two-level, within the “metabolism” pathway, subcategories such as “carbohydrate metabolism,” “amino acid metabolism,” “Metabolism of cofactors and vitamins,” and “Lipid metabolism” were significantly different between the two groups (Fig. S3F;* P* < 0.05). Subsequently, we analyzed the metabolic pathways at third-level. The results revealed that 7 carbohydrate metabolism pathways were significantly enriched in the CrPyr group, including “C5-branched dibasic acid metabolism,” “Pyruvate metabolism,” “Glycolysis/Gluconeogenesis,” “Starch and sucrose metabolism,” “Pentose phosphate pathway,” “Butanoate metabolism,” and “Propanoate metabolism” (Fig. 6A;* P* < 0.05). Additionally, five amino acid metabolism pathways (“Glutathione metabolism,” “d-Glutamine and d-glutamate metabolism,” “lysine degradation,” “Arginine biosynthesis,” and “Glycine-serine-threonine metabolism”) were also significantly enriched in the CrPyr group (Fig. 6B;* P* < 0.05). Consistently, comparative analysis of differentially enriched metabolic pathways between in vivo and in vitro datasets, revealed five pathways that were significantly elevated exclusively in the CrPyr group: d-glutamine and d-glutamate metabolism, glutathione metabolism, pyruvate metabolism, butanoate metabolism, and propanoate metabolism (Fig. S5).

#### Rumen metabolites as determined by targeted energy-metabolomics profiling of in vitro fermentation

A targeted metabolomic screen identified 30 metabolites linked to energy metabolism. Among them, glutamate, d-fructose-6-phosphate, succinate, oxaloacetate, l-lactate, and ATP were significantly elevated in the CrPyr group (Fig. 6D; *P* < 0.05). Notably, the upregulated intermediates d-fructose-6-phosphate, succinate, oxaloacetate, and l-lactate occupy key nodes in the VFA biosynthetic network (Fig. 6E). Integrating these metabolites with the corresponding pathways identified four enzymatic steps that were transcriptionally enriched under CrPyr supplementation: K01810 (*GPI*, *pgi*; d-glucose-6-phosphate → d-fructose-6-phosphate), K00174 (*korA*/*oorA*/*oforA*; pyruvate → acetyl-CoA), K01026 (*pct*; acetyl-CoA → butanoyl-CoA), and K01034 (*atoD*; acetyl-CoA → acetate). Cross-verification of these findings with the in vivo data (Fig. 3B) revealed a consistent enrichment of K00174 in the CrPyr group, supporting the hypothesis that CrPyr enhances the conversion from pyruvate to acetyl-CoA. Furthermore, ruminal ATP level was significantly higher in the CrPyr group (Fig. 6D). Subsequent profiling of genes involved in ATP synthesis, through the ETP route, demonstrated a selective upregulation of V/A-type ATPase subunits (Fig. S6A). In both in vitro and in vivo CrPyr datasets, 6 genes encoding V/A-type ATPase subunits, including *ATPVI*, *ATPVK*, *ATPVD*, *ATPVE*, *ATPVC*, *ATPVB* and *ATPVA*, were consistently upregulated (Fig. S6C). Moreover, glutamate is a key intermediate in GSH biosynthesis, and its increased abundance align with the enrichment of d-glutamine and d-glutamate metabolism as well as glutathione metabolism.

### Experiment 3: effects of CrPyr on colonic microbiota and glutathione synthesis in RMT mice

**Fig. 7:**
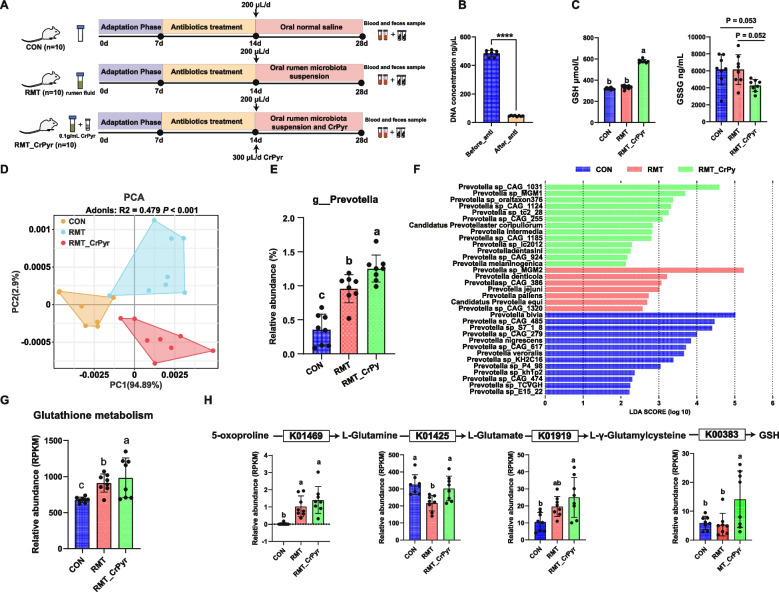
Effects of CrPyr on serum GSH, colonic *Prevotella*, and glutathione metabolism in RMT mice. **A** Study design diagram of the rumen fluid transplantation experiment. **B** Concentration of fecal DNA in mice before and after antibiotic treatment. **C** Levels of serum GSH and GSSG in the three groups. **D** Principal component analysis (PCA) of *Prevotella* at the species level. **E** Differential analysis of *Prevotella* at the genus level. **F** LEfSe analysis of differences in *Prevotella* at the species level, with LDA > 2. **G** Changes in the abundance of glutathione metabolism. **H** Changes in KOs related to GSH synthesis. Differences among the three groups were analyzed using one-way analysis of variance (ANOVA). Different lowercase letters indicate significant differences. ^a,b,c^*P* < 0.05. *n* = 8

The experimental design for Experiment 3 is depicted in Fig. 7A. To confirm the successful establishment of pseudo-germ-free mice, the fecal DNA content was measured before and after antibiotic treatment. Fecal DNA levels were significantly reduced following antibiotic treatment compared to the pre-treatment levels (Fig. 7B;* P* < 0.05), confirming successful establishment of the pseudo-germ-free mouse model. Based on the results from Experiments 1 and 2, we focused on changes in the abundance of *Prevotella* and the glutathione metabolism pathways in Experiment 3. Serum analysis revealed that CrPyr significantly increased GSH levels in RMT mice (*P* < 0.05) and showed a trend toward decreasing glutathione disulfide (GSSG) levels (*P* = 0.052; Fig. 7C). Constructing a gene set based on *Prevotella *sp. and analyzing it using Bray–Curtis dissimilarity showed distinct separations among the three groups (Fig. 7D; PERMANOVA *P* < 0.001). Subsequent differential analysis at the genus level indicated that CrPyr significantly enhanced the abundance of *Prevotella* in the colons of RMT mice (Fig. 7E;* P* < 0.05). At the species level, LEfSe analysis with an LDA score > 2 identified 33 *Prevotella* species (Fig. 7F;* P* < 0.05). However, comparison of differentially abundant bacteria from Experiment 1 and Experiment 2 revealed no common changes at the species level. The observed differences may be attributed to variations in diet composition and gut environment. In terms of metabolic pathways, the glutathione metabolism pathway was significantly enriched in the CrPyr-treated group (Fig. 7G;* P* < 0.05), consistent with the results from Experiment 1 and Experiment 2. Further identification of genes related to GSH synthesis showed that K01469 (*OPLAH*, *OXP1*, and *oplAH*), K01425 (*glsA*, and *GLS*), K01919 (g*shA*), and K00383 (*GSR*, and *gor*) were significantly enriched in the CrPyr group (Fig. 7H). This is consistent with the results of serum GSH.

## Discussion

Alleviating stress in newly received cattle is a major issue confronting the beef cattle industry. Cattle of any age experience stress before and after transportation, which is induced by a multitude of factors. Food deprivation during transport increases the risk of energy depletion, hunger, and hypoglycemia, especially in young calves with limited body fat reserves [1]. Although prior research has demonstrated some positive effects of additives such as probiotics [40], flavorings [41], and molasses [42] on mitigating stress in newly received cattle, a critical gap remains. Specifically, these studies have largely overlooked the potential of energy supplementation to alleviate stress. The significant workload, the complexity of clinical and laboratory outcomes, and the challenge of managing calves that are already under stress have further hindered a comprehensive exploration of this approach. Given the crucial role of energy metabolism in stress response, there is a pressing need to investigate additives that can directly address energy deficits in stressed cattle. Our study fills this gap by evaluating the impact of CrPyr, an additive with known energy-supplying properties, on stress regulation in newly received cattle on day 16 post-arrival. We explored its potential mechanisms through a comprehensive approach, including multi-omics analyses to provide an overview of the biological changes, followed by in vitro experiments to validate the findings and elucidate the specific pathways involved. Additionally, we conducted RMT experiments in mice to further investigate the mechanisms of stress regulation through which CrPyr regulates stress.

Hematological parameters can intuitively reflect the host’s stress and health status. In this study, we explored the effects of CrPyr on newly received cattle by examining serum hormone levels, antioxidant enzyme activities, and inflammatory factor levels. COR and ACTH are biomarkers of stress in transported animals. When animals are subjected to stress, the HPA axis and/or the sympathetic nervous system are activated, regulating the secretion of corresponding hormones by endocrine glands, primarily manifested as increased levels of ACTH and COR [43]. In our study, the serum levels of COR and ACTH in the CrPyr group were significantly reduced (Fig. 2B), indicating the potential of CrPyr to alleviate stress in newly received cattle. However, the specific mechanisms of action of CrPyr require further investigation. Serum ACTH and COR levels are positively correlated with the degree of oxidative stress [44]. In cattle, long-distance transportation promotes oxidative stress by disrupting the homeostasis of oxidants and antioxidants. This can subsequently initiate adverse inflammatory responses that are detrimental to overall health [8]. MDA is an important marker of oxidative stress. Our results showed that the serum MDA content in the CrPyr-treated group was significantly lower than that in the control group, while the activities of SOD and GSH-Px were significantly increased (Fig. 2B). This suggests that CrPyr enhances the host’s antioxidant capacity, which may be a reason for the decreased levels of COR and ACTH. Consistent with this, the levels of anti-inflammatory factors (IL-4) were upregulated, while the levels of pro-inflammatory factors (IL-1β, IL-6, and TNF-α) were downregulated (Fig. 2B), indicating that the inflammatory response in the host was alleviated. Based on the above results, we can conclude that CrPyr alleviates stress in newly received cattle by enhancing the host’s antioxidant capacity and reducing inflammatory responses, thereby lowering COR and ACTH levels. However, the mechanism by which how CrPyr enhances the host’s antioxidant capacity remains unclear. Given that the rumen microbiome plays a crucial role in host metabolism and can influence antioxidant status, we hypothesized that CrPyr might exert its effects through modulating the rumen microbiota and its associated metabolic pathways. We employed rumen metagenomics and metabolomics to explore this hypothesis and elucidate the underlying mechanisms.

*Prevotella*, a dominant genus in the rumen, is known for its metabolic versatility and ability to degrade complex carbohydrates, proteins, and amino acids [45, 46]. CrPyr supplementation provides both pyruvate and creatine substrates that *Prevotella* can use to enhance metabolic efficiency and biomass [25]. *Prevotella ruminicola* 23 has complex metabolic networks for nitrogen assimilation, and suitable carbon and nitrogen sources derived from CrPyr that could directly fuel its growth [47]. The presence of extensive polysaccharide utilization loci and carbohydrate-active enzymes in *Prevotella* further supports their adaptability to diverse nutrient sources [48]. Future studies should investigate whether *Prevotella* possesses specific transporters or enzymatic pathways for CrPyr or its metabolites, which could confer a direct growth advantage. Oxidative stress has been implicated as a potential pathogenic mechanism in various diseases associated with gut microbiome dysbiosis [49, 50]. Sun et al. [51] discovered that under oxidative stress conditions, gut microbiota tend to utilize *Ruminococcaceae* bacteria to synthesize GSH to combat stress. These findings indicate that gut microbiota plays a significant role in the host’s antioxidant capacity. In cattle subjected to long-distance transportation, the rumen microbiota undergoes substantial changes, including a significant decline in *Prevotella* abundance [6, 7]. *Prevotella* is the most abundant beneficial bacterium in the rumen, and its abundance changes can significantly impact host health. It has been reported that *Prevotella histicola* can improve cognitive impairments in rats with vascular dementia and reduce oxidative stress [52]. Furthermore, oxidative stress stimulates *Prevotella intermedia* to produce an adaptive response by altering the protein abundance in oxygen-adapted cells [53]. These findings suggest a close relationship between *Prevotella* and the host’s oxidative stress status. In the present study, CrPyr treatment significantly increased the abundance of *Prevotella* in the rumen of newly received cattle. Through in vivo and in vitro experiments, we identified six bacteria that increased simultaneously, including *P*. *brevis*, *P*. sp.* P5*-*50*, *P*. sp.* BP1*-*145*, *P*. sp.* BP1*-*148*, and *P*. sp.* AM42*-*24* (Fig. S5). Beyond changes in abundance, the functional characteristics of these *Prevotella* species warrant particular attention. *P*. sp.* P5*-*50*, *P*. sp. *BP1*-*145*, *P*. sp.* BP1*-*148*, and *P*. sp.* AM42*-*24* shares key roles in ruminal carbohydrate metabolism. On the one hand, during the degradation of polysaccharides, these strains produce short-chain fatty acids (including acetate, propionate, and butyrate), which serve as vital energy sources for ruminants and help maintain normal rumen function. On the other hand, these *Prevotella* strains interact synergistically with other microbes (such as *Fibrobacter*) in the rumen microbial community, promoting the degradation of plant fibers and thereby enhancing rumen fermentation efficiency [46, 54]. Additionally, Zhang et al. [55] isolated *P*. *brevis* from ruminal fluid and demonstrated, under axenic culture conditions, that this strain not only significantly increases the concentrations of acetate, propionate, and succinate, but also couples electron transport to ATP synthesis via a Na⁺-translocating Rnf complex, thereby accomplishing ETP. Additionally, they reported that the Rnf reaction simultaneously oxidizes NADH and reduces NAD⁺. This NAD⁺, together with the NADPH generated through fermentative redox balancing, feeds glutathione reductase and maintains a reduced GSH/GSSG ratio. Consequently, this ETP-driven cofactor recycling not only reduces auto-oxidation of excess NADPH (a major source of reactive oxygen species) but also enhances the antioxidant capacity of* P*. *brevis*, while continuing to produce beneficial VFA. Further analysis of microbial functions revealed that five metabolic pathways were significantly altered in the CrPyr treatment group through in vivo and in vitro experiments (Fig. S4), including d-glutamate and d-glutamine metabolism, glutathione metabolism, pyruvate metabolism, butyrate metabolism, and propionate metabolism. Among them, d-glutamate and d-glutamine metabolism and glutathione metabolism are involved in the regulation of host oxidative stress. Specifically, glutamate synthesized via the d-glutamate and d-glutamine metabolism pathways can enter the glutathione metabolism pathway to produce GSH, which plays a crucial role in the removal of various reactive species [56]. Concurrently, changes in rumen metabolite glutamate content are in line with these findings (Figs. 4D and 6D). Despite the significant differences in physiological characteristics and digestive systems between ruminants and non-ruminants, a previous study found that the composition of rumen microbiota is similar to that of the mouse colonic microbiota [37]. Additionally, many scholars have used RMT mice to verify the mechanisms through which rumen microbiota affect the host [57, 58]. In our study, although the abundance of *Prevotella* at the genus level in the CrPyr group was significantly increased (Fig. 7E), no *Prevotella* species that changed consistently varied in both experiment 1 and experiment 2. These findings suggest that the effects of the rumen microbiota on the host are likely driven by multiple species or broader community functions rather than a single specific species. This highlights the complexity and functional redundancy of microbial communities, indicating that future research should consider the overall structure and function of the microbial community, rather than focusing only on changes in individual species. Additionally, under the RMT model, glutathione metabolism was significantly enriched in the CrPyr group (Fig. 7G), and the abundance of enzymes related to GSH synthesis, such as 5-oxoprolinase (K01469), glutaminase (K01425), glutamate-cysteine ligase (K01919), and glutathione reductase (K00383), was the highest in the CrPyr group, further indicating that CrPyr positively regulates glutathione metabolism. Additionally, the changes in the serum metabolites GSH and GSSG in mice were in line with these findings (Fig. 7C). In summary, these results support the hypothesis that CrPyr can enhance the antioxidant capacity of newly received cattle and help the host in resisting stress by regulating the abundance of *Prevotella* to mediate GSH synthesis. Future studies should focus on identifying the specific *Prevotella* species regulated by CrPyr and to elucidate the underlying molecular mechanisms.

Furthermore, *Prevotella* plays a crucial role in the synthesis of VFA. This genus can utilize starch and protein to produce succinate and acetate, making it one of the most abundant core genera in the rumen [59]. Dong et al. [60] reported that increasing the concentration of VFA in the rumen can alleviate weaning stress in young ruminants. Moreover, enhanced VFA synthesis capacity helps Tibetan sheep adapt to nutritional stress during the cold seasons at high altitudes [61]. These findings suggest that increasing VFA levels can provide the host with additional energy support, thereby enhancing its stress resistance. In our in vivo and in vitro experiments, the CrPyr treatment group exhibited the highest levels of VFA (acetate, propionate, and butyrate). Besides, the metabolic pathways associated with VFA synthesis (pyruvate, butyrate, and propionate metabolism) were significantly enriched. This indicates that CrPyr may promote VFA synthesis by modulating the abundance of *Prevotella*. Additionally, the key enzyme involved in the conversion of pyruvate to acetyl-CoA, 2-oxoglutarate synthase (K00174), was most abundant in the CrPyr group, further supporting this finding. Concurrently, changes in rumen metabolites also confirmed this: oxaloacetate, succinate, and lactate, which are closely related to propionate synthesis, were significantly elevated in the CrPyr group. These results demonstrate that CrPyr promotes VFA synthesis by modulating the abundance of *Prevotella*, providing the host with additional energy support and enhancing its stress resistance. However, the mechanisms through which CrPyr increases *Prevotella* abundance remain to be investigated.

ATP serves as the energy currency of microbial life, primarily generated through SLP and ETP [62]. Under anaerobic conditions, SLP promotes ATP synthesis through glycolysis, during which high-energy phosphate groups are directly transferred from substrate molecules to ADP [63]. In contrast, ETP utilizes the electrochemical gradient generated by the respiratory chain to drive efficient ATP synthesis through ATP synthase, a more effective but rate-limiting step [64, 65]. In our experiments, no significant changes were observed in SLP, whereas the abundance of V/A-type ATP synthase in the ETP pathway was significantly increased in the CrPyr group. Additionally, seven genes regulating V/A-type ATP synthase (*ATPVI*, *ATPVK*, *ATPVD*, *ATPVE*, *ATPVC*, *ATPVB*, and *ATPVA*) showed similar trends (Fig. S5). These findings suggest that CrPyr may enhance ATP synthesis in the rumen of newly received cattle through the ETP pathway. This hypothesis is further supported by the significant increase in rumen ATP content observed in both in vivo and in vitro experiments. Concurrently, differential metabolites in the rumen were identified and mainly enriched in purine metabolism (d-ribose-5-phosphate, cGMP, and guanine) and pyrimidine metabolism (5-CMP, and Uridine-5-monophosphate). Purine and pyrimidine metabolism provide nucleotide substrates for microbial proliferation [66]. In summary, CrPyr is associated with an increased genetic potential for ETP-driven ATP synthesis, providing an energetic basis for *Prevotella* proliferation, and enriches purine and pyrimidine metabolism pathways, supplying essential nucleotides for microbial growth. It should be emphasized that metagenomics only provides correlative evidence based on gene abundance; it does not directly demonstrate higher enzymatic activity, enhanced proton gradient formation, or an actual shift from SLP to ETP. Transcript abundance, protein expression levels, membrane potential measurements, or in vitro ATPase activity assays are required to functionally validate this hypothesis.

## Limitations and future directions

In this study, we discovered that CrPyr can mitigate stress in newly received cattle by regulating the abundance of *Prevotella* in the rumen. However, this study has several limitations. First, although enrichment of ATP synthesis, purine, and pyrimidine metabolic pathways, together with increase *Prevotella* abundance, was observed in the CrPyr group, the direct interactions among these elements require further investigation. Second, both in vitro experiments and RMT trials indicated that CrPyr can regulate glutathione metabolism and promotes GSH synthesize by increasing the abundance of *Prevotella*, thereby combating oxidative stress in newly received cattle. However, these microbiota-associated changes were identified primarily at the genus level. Future studies should use targeted cultivation or microbial monoculture transplantation techniques to confirm the specific roles of key microbial species and further explore the regulatory relationship between *Prevotella* and glutathione.

Furthermore, mice lack a rumen and differ anatomically and physiologically from ruminants; however, accumulating evidence supports the use of murine models to functionally validate rumen-derived microbiota. Previous studies have successfully demonstrated that RMT from diseased ruminants (with SARA or mastitis) to antibiotic-treated or germ-free mice can reproduce relevant host phenotypes, including colonic inflammation, immune dysregulation, and microbial dysbiosis [9, 37, 58]. These findings suggest that, despite interspecies differences, key microbial, host interactions, particularly those involving immune and inflammatory pathways, are at least partially conserved across mammalian hosts. In this study, we utilized a mouse model to explore the systemic effects of rumen microbiota modulated by CrPyr supplementation. This approach provides valuable mechanistic insights under controlled conditions; however, it also introduces limitations related to translational relevance. The absence of a rumen environment, differences in gut pH, microbial colonization dynamics, and immune architecture may limit the translational relevance of our findings to ruminants. Consequently, the mouse model serves as a useful tool for hypothesis generation and preliminary functional screening; however, the results should be carefully interpreted with appropriate caution. Importantly, to definitively exclude the possibility that CrPyr alleviates stress through directly targeting host tissues, microbiota-depletion/re-colonization experiments are required. Recipients rendered pseudo-germ-free by broad-spectrum antibiotics should receive either a *Prevotella*-enriched consortium (isolated from CrPyr-treated donors) or the corresponding *Prevotella*-free microbiota; comparison of stress biomarkers and GSH status between the two groups would clarify whether *Prevotella* is a required intermediary for CrPyr’s anti-stress effect.

Addressing these limitations in future research will establish a more comprehensive theoretical foundation for the stress-mitigating effects of CrPyr in newly received cattle. It will facilitate the rational development of targeted interventions to optimize rumen function and improve livestock productivity.

## Conclusion

**Fig. 8:**
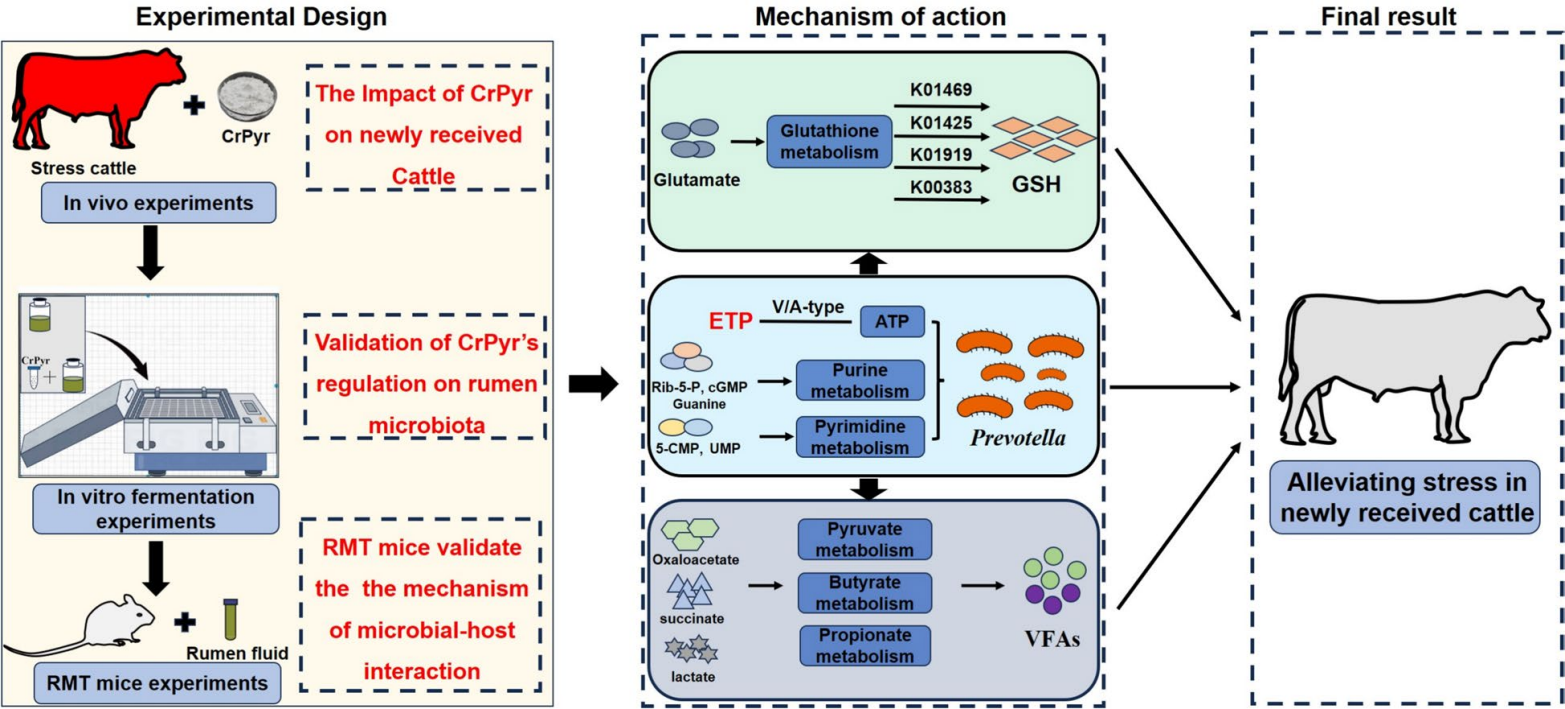
Full text of the overview summary.CrPyr increases the abundance of *Prevotella*, a key rumen bacterium involved in volatile fatty acid (VFA) production, and enriches metabolic pathways related to energy metabolism (ATP synthesis, pyruvate metabolism) and antioxidant defense (glutathione metabolism). In vitro experiments as well as RMT studies in mice further validate these findings, demonstrating that CrPyr promotes VFAs synthesis and enhances ATP production via the electron transport phosphorylation (ETP) pathway. K01469 (*OPLAH*, *OXP1*, *oplAH*; 5-oxoprolinase), K01425 (*glsA*, *GLS*; glutaminase), K01919 (*gshA*; glutamate-cysteine ligase), and K00383 (*GSR*, gor; glutathione reductase)

This study provides a comprehensive understanding of the mechanisms by which CrPyr alleviates stress in newly received cattle through multi-omics approaches, in vitro and in vivo experiments, and RMT studies (Fig. 8). Our findings reveal that CrPyr mitigates stress by regulating the abundance of *Prevotella* in the rumen, which in turn enhances glutathione metabolism and VFA synthesis, providing additional energy support to the host. Additionally, CrPyr promotes ATP synthesis through the ETP pathway and enriches purine and pyrimidine metabolism pathways, supplying essential nucleotide substrates that support microbial proliferation and functional activity. These findings highlight the potential of CrPyr as a nutritional intervention to optimize rumen function and enhance livestock productivity by alleviating stress in newly received cattle. Future research should focus on identifying the specific *Prevotella* species regulated by CrPyr and elucidating the molecular mechanisms underlying the regulatory relationship between *Prevotella* and glutathione metabolism.

## Supplementary Information

Supplementary Material 1: Supplementary Fig. S1–S6.

Supplementary Material 2: Supplementary Table S1–S16.

## Data Availability

The datasets analyzed during the current study are available from the corresponding author on reasonable request. Metagenome sequence data associated with this project have been deposited in the NCBI Short Read Archive database (BioProject ID: PRJNA1310242 (https://www.ncbi.nlm.nih.gov/search/all/?term=PRJNA1310242), BioProject ID: PRJNA1310418 (https://www.ncbi.nlm.nih.gov/search/all/?term=PRJNA1310418), BioProject ID: PRJNA1320625 (https://www.ncbi.nlm.nih.gov/search/all/?term=PRJNA1320625). The metabolomics data have been deposited in the National Center for Bioinformation under accession number OMIX013787 (https://ngdc.cncb.ac.cn/omix/release/OMIX013787).

## Abbreviations

ACTH: Adreno cortical tropic hormone
CAZymes: Carbohydrate-active enzymes
CK: Creatine kinase
COR: Cortisol
GSH-PX: Glutathione per-oxidase
IL-1β: Interleukin-1β
KEGG: Kyoto encyclopedia of genes and genomes
MCP: Microbial crude protein
MDA: Malondialdehyde
PC: Phosphatidylcholine
SOD: Superoxide dismutase
T-AOC: Total antioxidant capacity
TNF-α: Tumor necrosis factor alpha
VFA: Volatile fatty acid
VIP: Variable importance in the projection
RA: Relative abundance
