# Supplementary material for: Rumen microbiota-associated stress alleviation by creatine pyruvate in newly received cattle: a multi-omics study
Source: Microbiome. 2026 Mar 5;14:114. doi: 10.1186/s40168-026-02365-1 (PMC13069763; doi:10.1186/s40168-026-02365-1)
Supplement: Supplementary file 2 — Supplementary Material 1: Supplementary Fig. S1–S6. [file 40168_2026_2365_MOESM1_ESM.docx]

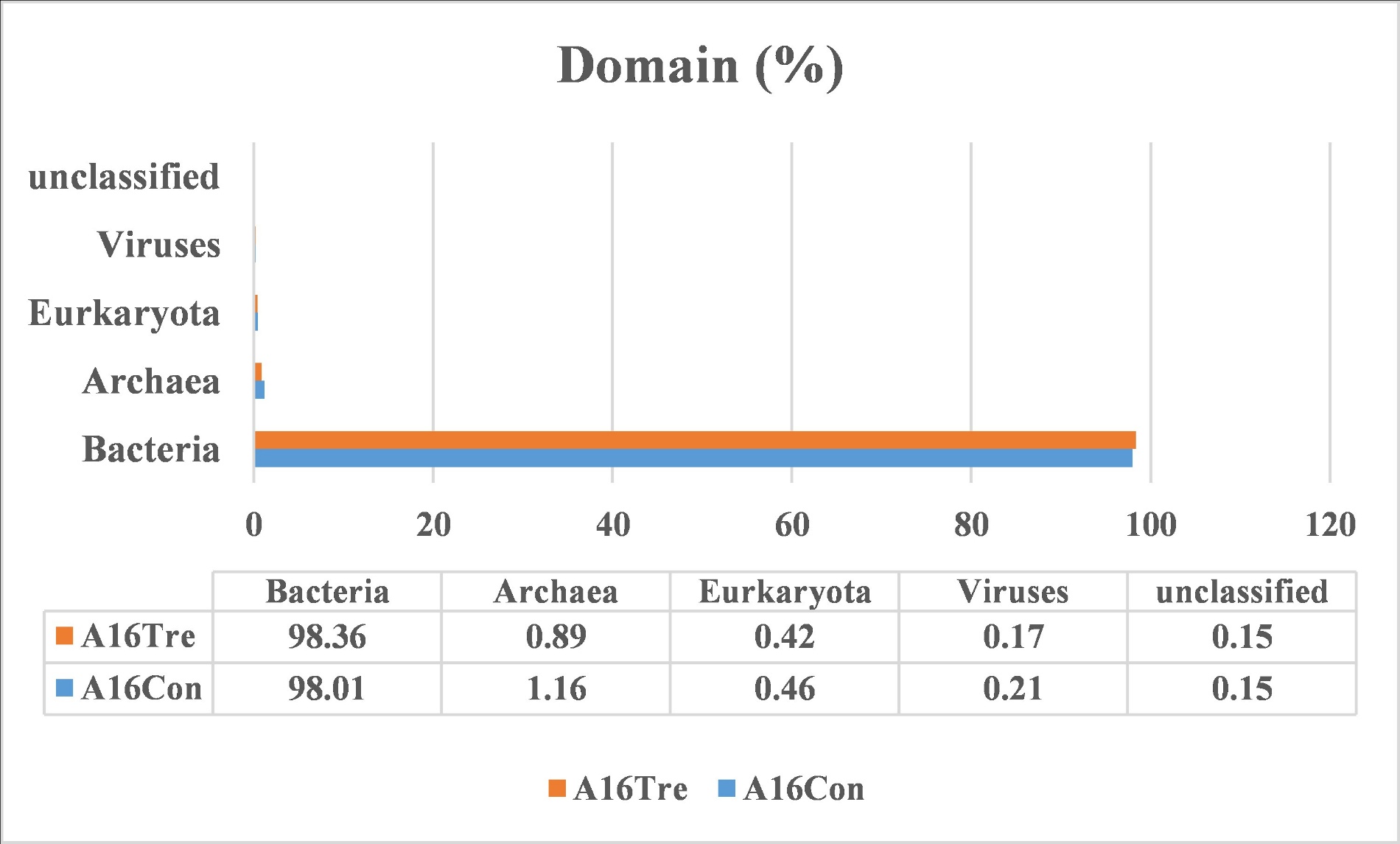


**Fig. S1** Comparison of microbial domains in newly received cattle between the two groups


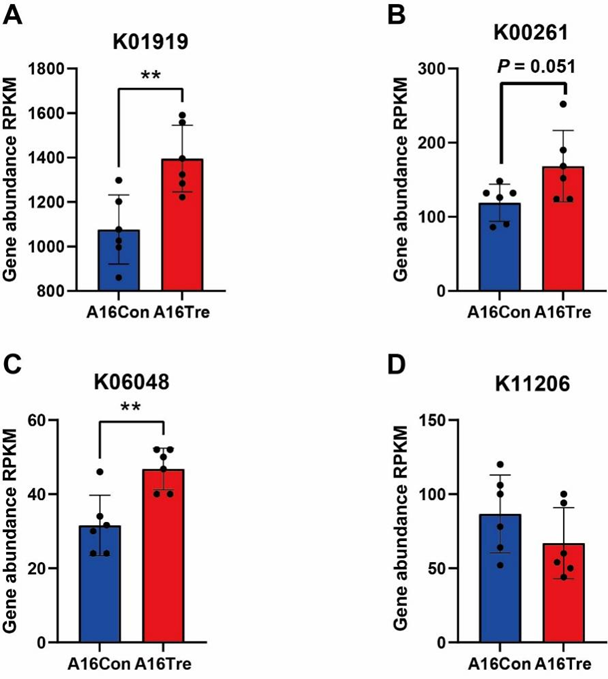


**Fig. S2** Expression of genes involved in glutamate biosynthesis**. A** K01919: deaminated glutathione amidase [EC:3.5.1.128]; **B** K00261: glutamate dehydrogenase [EC:1.4.1.3]; **C** K06048: glutamate-cysteine ligase/carboxylate-amine ligase [EC:6.3.2.2]; **D** K11206 deaminated glutathione amidase [EC:3.5.1.128].


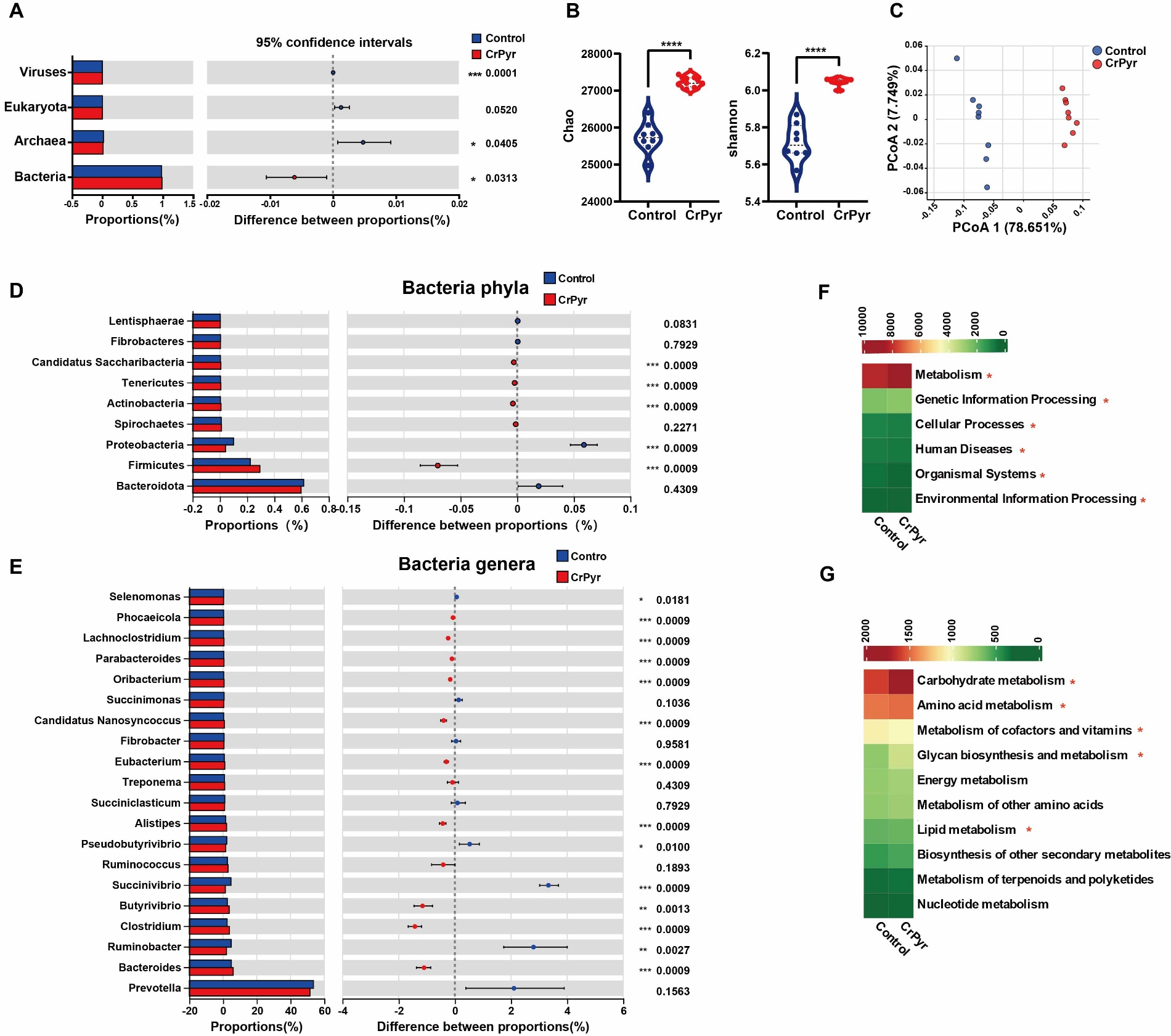


**Fig. S3** Comparison of microbial and function feature changes in the in vitro fermentation model between the two groups. **A** Comparison microbial domains level. **B** Bacteria alpha diversity. **C** Bacteria species level beta diversity. **D** Differential analysis of the top 10 bacteria at the phylum level. **E** Differential analysis of the top 20 bacteria at the genu level. **F** Level 1 function classification. **G** Metabolism level 2 function classification. Significantly different domains were tested by Wilcoxon rank-sum test with ^*^*P* value of < 0.05, ^**^*P* < 0.01, ^***^ *P* < 0.001.


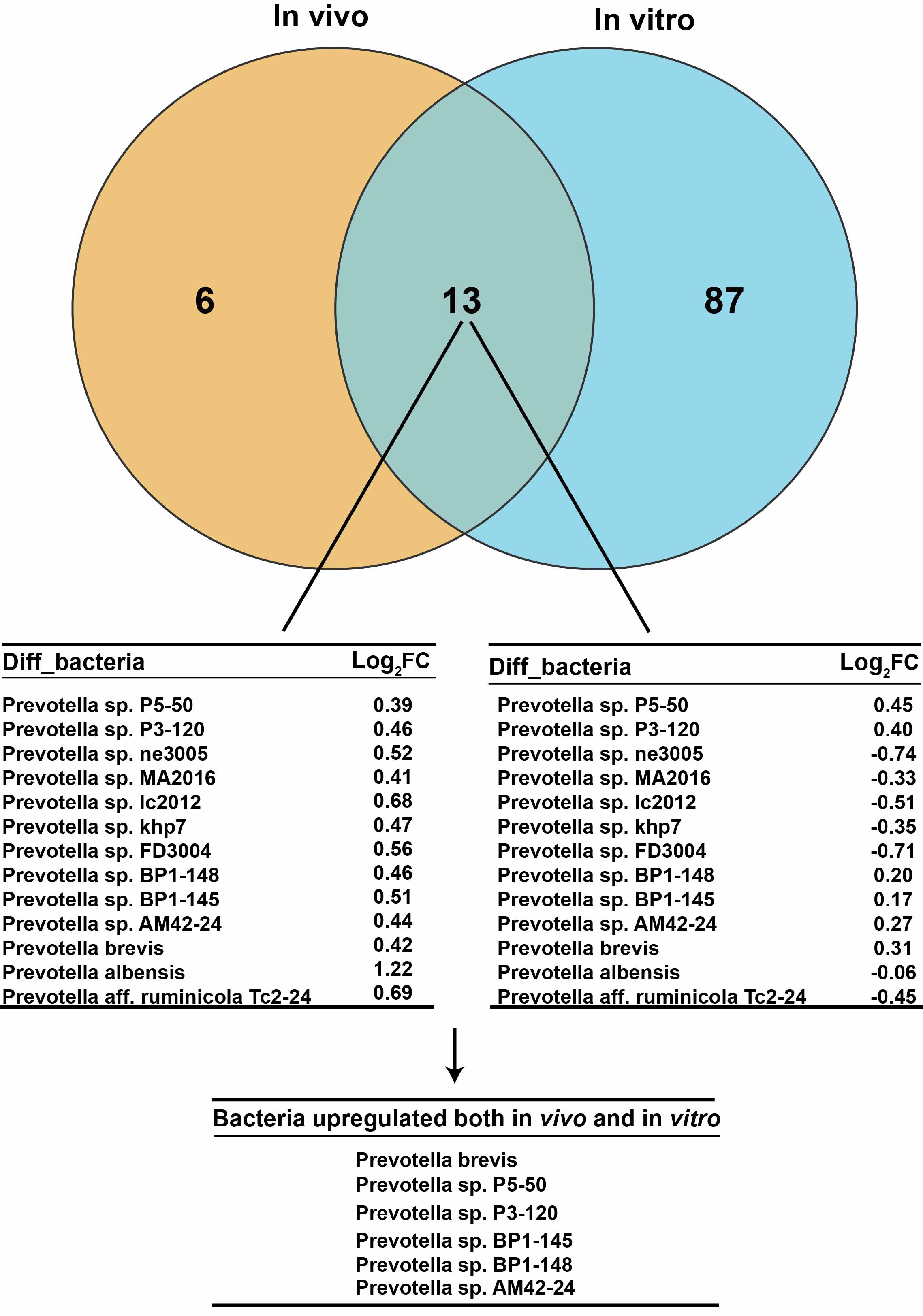


**Fig. S4** The number of common bacterial species differs between in vivo and in vitro conditions


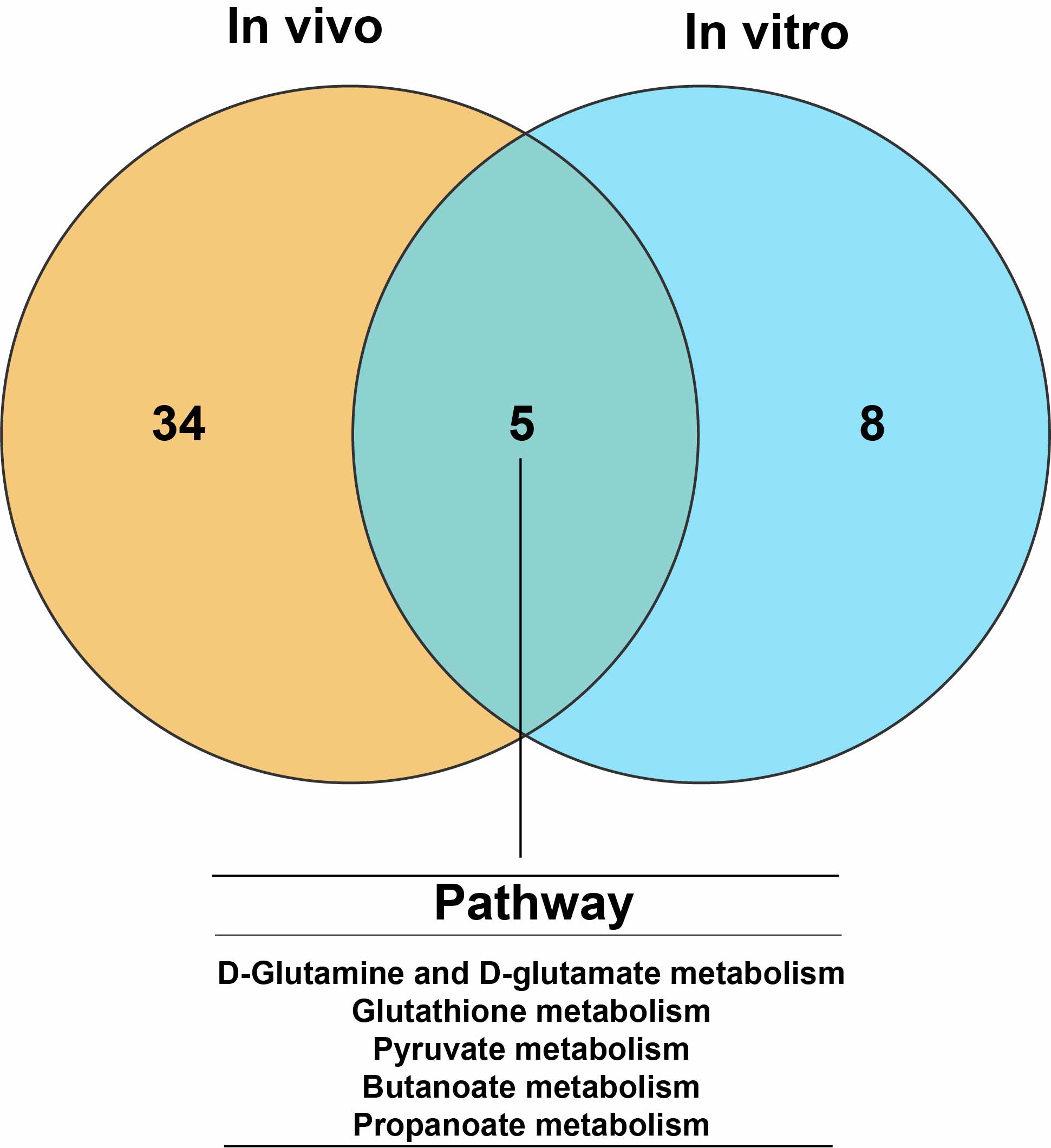


**Fig. S5** The number of common metabolic pathways differs between in vivo and in vitro conditions


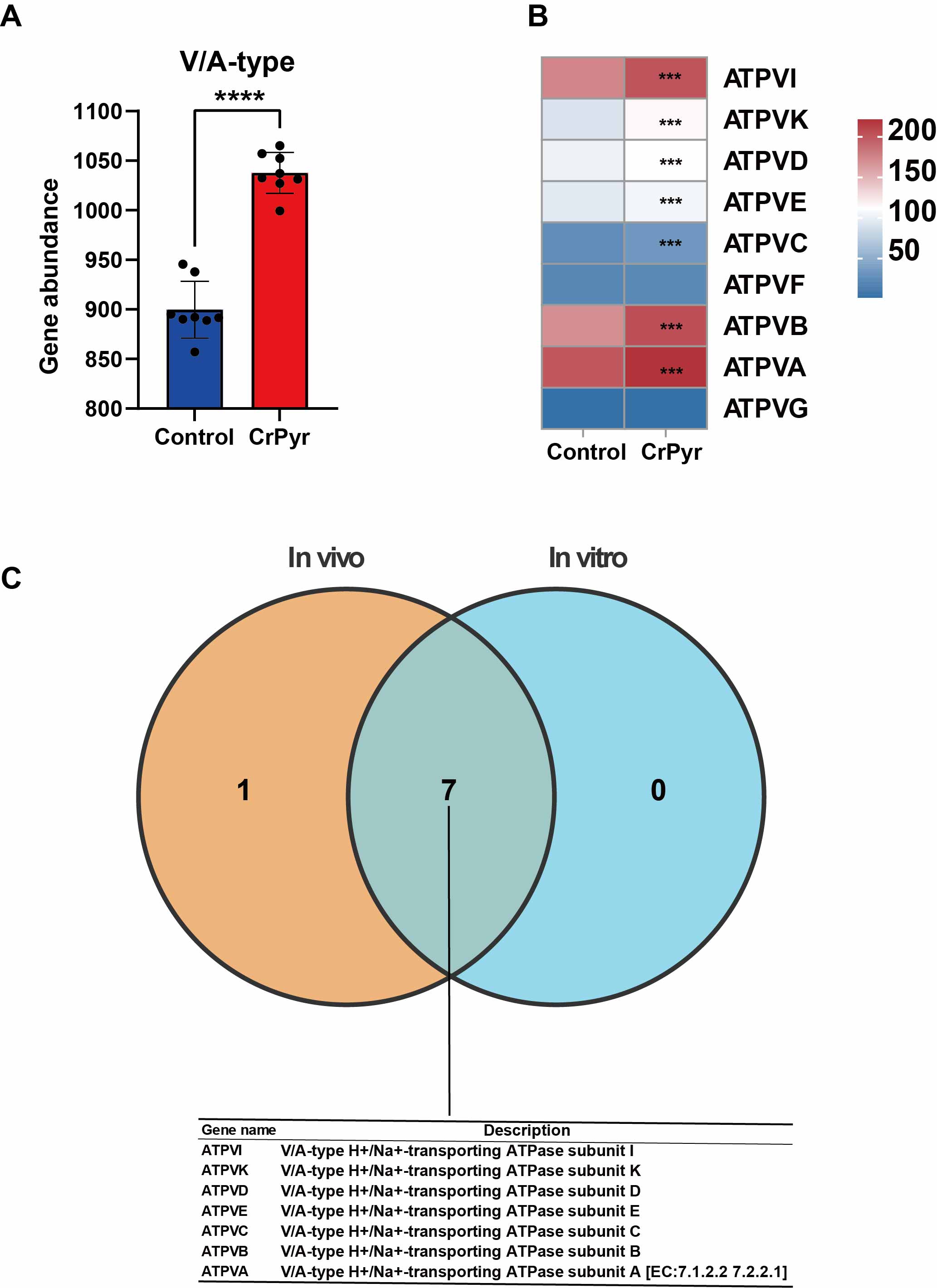


**Fig. S6** Effects of CrPyr on ATP synthase in an in vitro rumen fermentation model. **A** Total abundance of V/A-type ATP synthase. **B** Composition and differential analysis of V/A-type ATP synthase. **C** Upregulated ATP synthase in in vivo and in vitro models.
